# A FAP‐Targeted SMDC Platform Enables Synergistic Radionuclide–Chemotherapy with PET‐Guided Evaluation

**DOI:** 10.1002/advs.75150

**Published:** 2026-04-07

**Authors:** Ruitao Yang, Hongxin Li, Zexin Xu, Baocheng Chen, Ningjie Li, Yuhua Zhong, Kongzhen Hu

**Affiliations:** ^1^ Department of Nuclear Medicine Nanfang Hospital Southern Medical University Guangzhou China; ^2^ School of Pharmaceutical Sciences Southern Medical University Guangzhou China; ^3^ Department of Rehabilitation Medicine Nanfang Hospital Southern Medical University Guangzhou China

**Keywords:** cleavable linker, fibroblast activation protein (FAP), radio–chemo therapy, small molecule drug conjugates (SMDCs), theranostics

## Abstract

Fibroblast activation protein (FAP) is an attractive stromal target for cancer theranostics; however, developing agents that enable non‐invasive pharmacokinetic visualization while achieving effective combined radio‐chemotherapy remains challenging. Here we report an imaging‐enabled FAP‐targeted small molecule–drug conjugate (SMDC) platform that supports PET‐based pharmacokinetic visualization and synergistic radionuclide–chemotherapy using matched radiolabeled constructs. In this system, ^68^Ga‐labeled SMDCs enable positron emission tomography (PET) imaging to visualize in vivo biodistribution, whereas the corresponding ^177^Lu‐labeled analogues deliver combined radionuclide and cytotoxic therapy. By systematically comparing VC and GP cleavable linkers in DOTA‐containing MMAE conjugates, we establish a linker‐dependent structure–stability–efficacy relationship. FAPI‐46–based constructs retained nanomolar FAP affinity and enabled efficient enzyme‐triggered payload release while maintaining selective tumor accumulation in vivo as visualized by ^68^Ga‐PET. Therapeutic studies using ^177^Lu‐labeled conjugates produced pronounced tumor suppression (92–93% inhibition), outperforming either modality alone. Notably, the VC‐linked construct showed improved tolerability relative to the GP analogue. These findings establish an imaging‐guided strategy for optimizing FAP‐targeted radiochemotherapeutics and identify FAP‐O‐VC‐MMAE as a promising candidate for further translational development.

## Graphical abstract

A fibroblast activation protein (FAP)‐targeted small molecule–drug conjugate (SMDC) platform is developed to integrate PET imaging with synergistic radionuclide–chemotherapy. Linker chemistry governs stability, exposure, and tolerability, with the VC linker providing superior in vivo performance. This imaging‐enabled strategy establishes a structure–stability–efficacy framework for optimizing radiochemotherapeutic SMDCs.

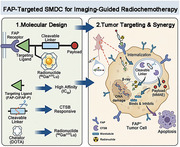

**Abbreviations**
ADC: Antibody‐Drug Conjugate
CAFs: Cancer‐Associated Fibroblasts
CTSB: Cathepsin B
DOTA: 1,4,7,10‐Tetraazacyclododecane‐1,4,7,10‐tetraacetic acid
FAP: Fibroblast Activation Protein
GP: Glycine‐Proline
MMAE: Monomethyl Auristatin E
PABC: para‐Aminobenzyloxycarbonyl
SMDC: Small Molecule‐Drug Conjugate
TGI: Tumor Growth Inhibition
VC: Valine‐Citrulline

## 1 Introduction

Fibroblast activation protein (FAP), a type II transmembrane serine protease, is overexpressed in more than 90% of epithelial malignancies [1, 2, 3], while being largely absent in most healthy adult tissues [4, 5, 6, 7]. Furthermore, FAP expression is primarily restricted to cancer‐associated fibroblasts (CAFs) within the tumor stroma [1, 2, 3], which provides a strong rationale for exploiting the tumor microenvironment as a target for both diagnostic imaging and therapeutic intervention [8]. Small‐molecule ligands capable of selectively binding tumor‐associated targets with high affinity have therefore attracted increasing interest as delivery vehicles for radionuclides, cytotoxic drugs, and optical probes [9, 10, 11, 12, 13, 14]. Compared with macromolecular carriers, low‐molecular‐weight ligands offer several advantages, including improved tumor penetration, rapid pharmacokinetics [15], reduced immunogenicity [16], and scalable synthesis [17]. Various small‐molecule FAP‐targeting ligands have been developed, including clinically established quinoline‐based FAP inhibitors (e.g., FAPI‐04) [18, 19, 20, 21] and high‐affinity cyclic peptides (e.g., FAP‐2286) [22]. Both classes have been successfully conjugated to radionuclides to generate diagnostic agents that demonstrate excellent tumor visualization and high tumor‐to‐background contrast in clinical studies, thereby validating FAP as a robust target for oncologic imaging.

Despite these encouraging diagnostic results, the therapeutic translation of FAP‐targeted radioligands remains challenging due to rapid clearance, insufficient tumor retention, and off‐target irradiation, which can restrict the deliverable therapeutic dose and increase toxicity risks [23]. Similar challenges are encountered in the development of FAP‐targeted small molecule–drug conjugates (SMDCs) [24]. A critical aspect of SMDC design lies in the selection of cleavable linkers that maintain sufficient systemic stability during circulation while enabling efficient intracellular release of cytotoxic payloads. Among the various linker systems reported for targeted drug delivery, cathepsin B–sensitive dipeptide linkers such as VC have been widely used in antibody–drug conjugates due to their favorable stability and efficient lysosomal cleavage [25, 26, 27]. GP linkers have also been explored in FAP‐targeted SMDC systems and exhibit distinct cleavage behavior in the tumor microenvironment [24]. However, the performance of such linkers may depend strongly on the overall molecular architecture of the conjugate, including the targeting ligand, payload, and auxiliary functional modules.

Another challenge in SMDC development is the lack of built‐in strategies for non‐invasive evaluation of pharmacokinetics and tumor targeting during preclinical optimization [28, 29, 30, 31]. Radiometal chelators such as 1,4,7,10‐tetraazacyclododecane‐1,4,7,10‐tetraacetic acid (DOTA) facilitate theranostic development by allowing the same molecular framework to incorporate either diagnostic radionuclides, such as gallium‐68 for positron emission tomography (PET) imaging, or therapeutic radionuclides, such as lutetium‐177 for targeted radionuclide therapy. This modular design offers an attractive approach for imaging‐guided SMDC development, enabling real‐time visualization of in vivo biodistribution and pharmacokinetics with PET imaging analogues, while therapeutic counterparts based on the same scaffold can be used to investigate radionuclide therapy and its potential synergy with intracellularly released cytotoxic agent [32, 33, 34, 35, 36, 37, 38].

**FIGURE 1:**
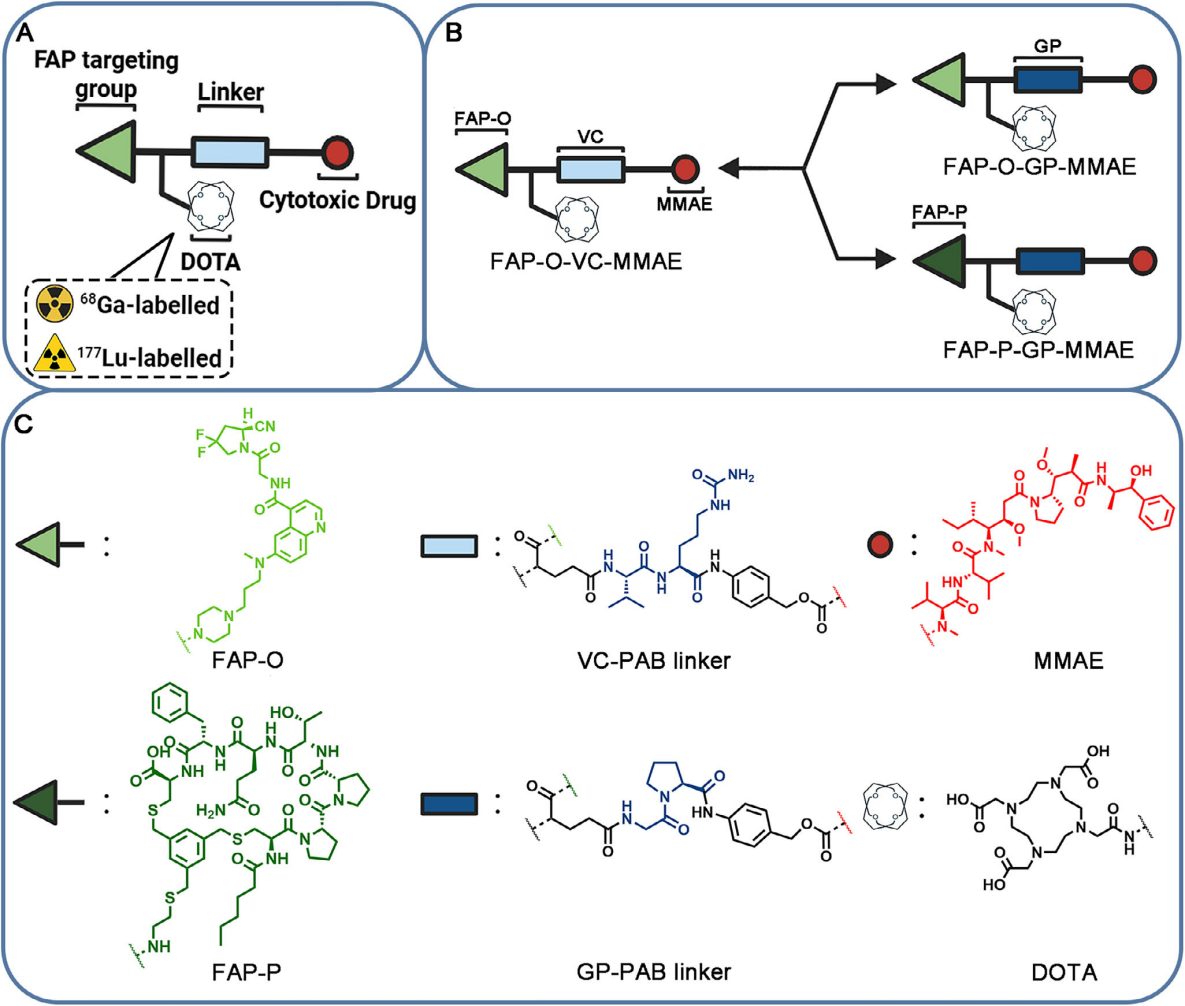
Design and composition of the FAP‐targeted theranostic SMDCs. (A) General SMDC architecture consisting of an FAP‐targeting moiety (arrow), a protease‐cleavable linker (rectangle), and the cytotoxic payload MMAE (circle), with an integrated DOTA chelator (octahedron) for ^68^Ga/^177^Lu radiolabeling. (B) Three candidate conjugates (FAP‐O‐VC‐MMAE, FAP‐O‐GP‐MMAE, and FAP‐P‐GP‐MMAE) generated by modular exchange of the targeting ligand (FAP‐O or FAP‐P) and linker (VC‐PABC or GP‐PABC). (C) Chemical structures of the core components, including the targeting ligands (FAP‐O and FAP‐P), cleavable linkers (VC‐PABC and GP‐PABC), cytotoxic payload (MMAE), and chelator (DOTA).

Based on these considerations, we designed an imaging‐enabled FAP‐targeted theranostic SMDC platform by integrating a radiometal chelator and the microtubule inhibitor monomethyl auristatin E (MMAE) into a single FAP‐binding scaffold. MMAE is a potent tubulin polymerization inhibitor widely used as a payload in targeted drug conjugates due to its high cytotoxic potency and well‐characterized intracellular mechanism of action. The modular architecture of the FAP‐targeted SMDCs and the design of the candidate conjugates are illustrated in Figure 1. Three representative conjugates were constructed to systematically evaluate the effects of targeting ligand class and linker chemistry within a unified molecular framework. In this system, matched radiolabeled constructs allow PET imaging using ^68^Ga‐labeled analogues and therapeutic evaluation using ^177^Lu‐labeled counterparts. Using this modular strategy, we systematically investigated the influence of targeting ligand class (quinoline‐based FAP inhibitor, FAP‐O, versus cyclic peptide ligand, FAP‐P) and linker chemistry (VC vs GP) on pharmacokinetics, metabolic stability, tumor targeting, and therapeutic efficacy. Through this comparative framework, we aim to establish a structure–stability–efficacy relationship for multifunctional FAP‐targeted SMDCs and to identify molecular design principles that enable effective imaging‐guided radiochemotherapeutic development.

## 2 Results and Discussion

### 2.1 Design, Synthesis, and Linker‐Dependent Stability of FAP‐Targeted SMDCs

**FIGURE 2:**
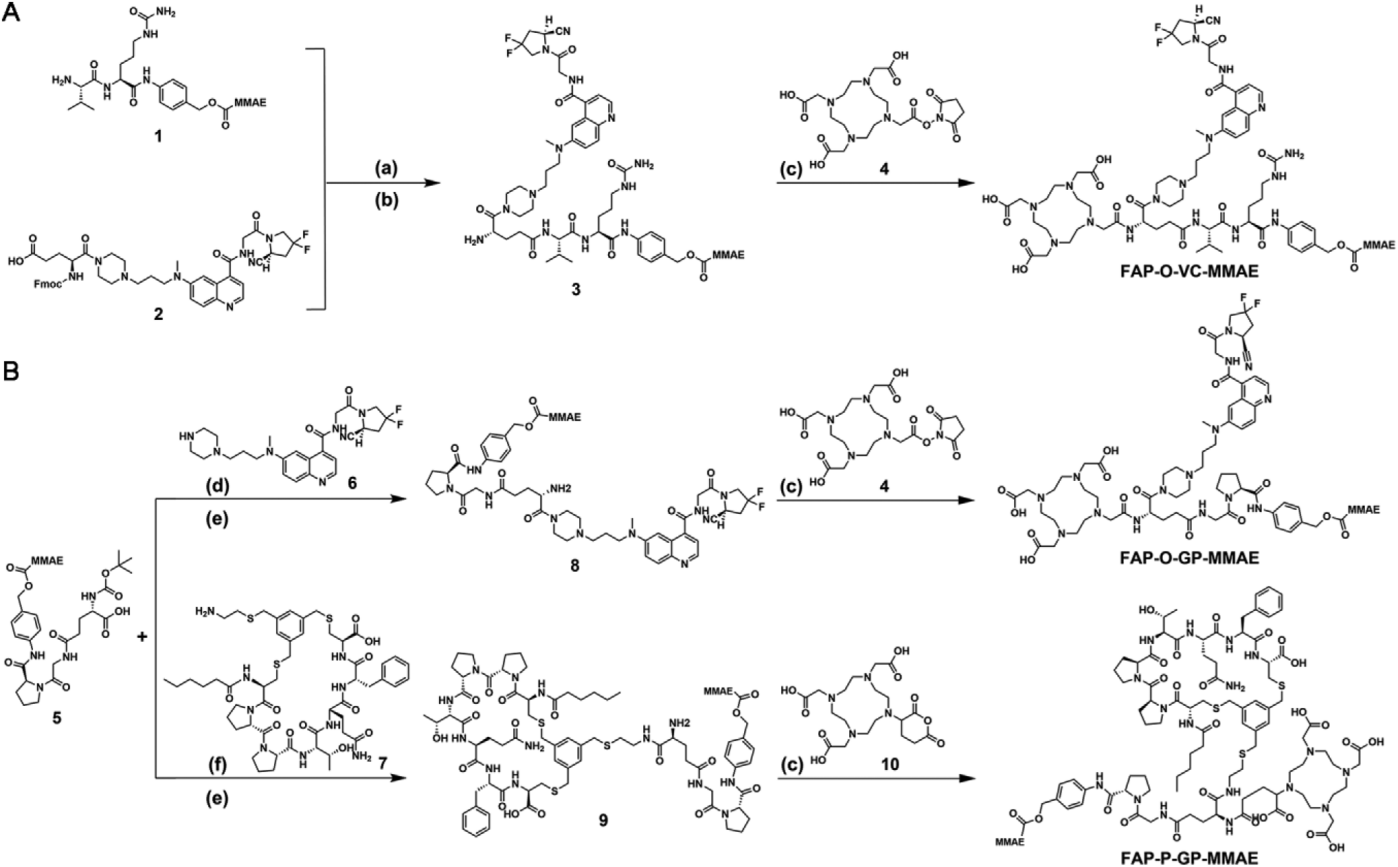
Schematic illustration of the modular assembly of FAP‐targeted SMDCs. (A) Assembly of FAP‐O‐VC‐MMAE. (B) Assembly of FAP‐O‐GP‐MMAE and FAP‐P‐GP‐MMAE. The cytotoxic payload is denoted as MMAE. Reagents and conditions: (a) EDCI, HOOBt, NMM, DMF; (b) 20% PIP/DMF; (c) DIEA, DMF; (d) HATU, DIPEA, DMF; (e) TFA; (f) TSTU, DIEA, DMF.

To enable PET visualization and radiochemotherapeutic evaluation within a unified molecular framework, we assembled three FAP‐targeted MMAE‐based SMDCs that differ by targeting vector class and cleavable dipeptide linker: FAP‐O‐VC‐MMAE, FAP‐O‐GP‐MMAE, and FAP‐P‐GP‐MMAE. These constructs were designed to systematically evaluate the influence of targeting ligand class (organic small‐molecule ligand FAP‐O vs cyclic peptide ligand FAP‐P) and linker chemistry (VC vs GP) on the pharmacological behavior of multifunctional SMDCs. A peptide‐based VC analogue was not included in the present study because early design considerations indicated reduced structural tolerance of the peptide scaffold after simultaneous incorporation of the chelator and hydrophobic payload, and we therefore prioritized a representative peptide‐linked comparator within the scope of this initial platform study. All compounds were obtained by multi‐step solid‐phase synthesis (Figure 2), purified to >97% by HPLC, and confirmed by mass spectrometry (routes and characterization are provided in Scheme S1, S2 and Figures S1–S12).

**FIGURE 3:**
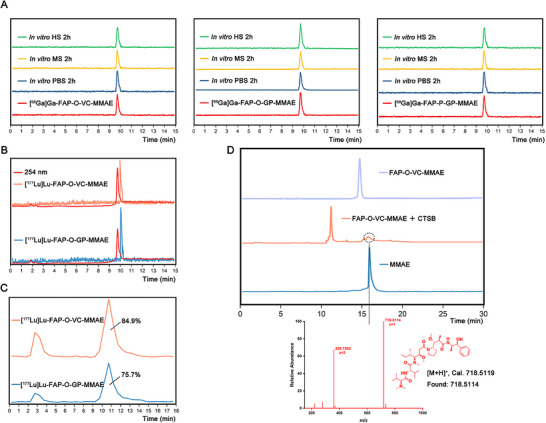
Stability and enzymatic cleavage of SMDCs. (A) Representative radio‐HPLC chromatograms showing the in vitro stability of the three [^68^Ga]Ga‐labeled SMDCs after 2 h incubation in reaction mixture, phosphate‐buffered saline (PBS), mouse serum (MS), and human serum (HS). (B) Representative radio‐HPLC chromatograms of purified [^177^Lu]Lu‐labeled SMDCs, showing [^177^Lu]Lu‐FAP‐O‐VC‐MMAE (upper) and [^177^Lu]Lu‐FAP‐O‐GP‐MMAE (lower). Corresponding UV traces were recorded at 254 nm. (C) Representative radio‐HPLC chromatograms showing the in vivo stability of [^177^Lu]Lu‐labeled SMDCs in mouse blood collected 24 h post‐injection (n = 3 mice per compound). (D) Cathepsin B (CTSB)‐mediated cleavage of FAP‐O‐VC‐MMAE monitored by HPLC (upper), with cleavage products identified by high‐resolution mass spectrometry (HRMS, lower).

All ligands were labeled with gallium‐68 under standardized conditions (105°C, 10 min), affording comparable radiochemical performance across the series, with non‐decay‐corrected yields of 53.3–54.8%, radiochemical purity >99%, and molar activities of 20.71–33.24 GBq/µmol (n = 5; Table 1). Lipophilicity differed modestly between the two organic‐ligand conjugates (logD = −0.58 ± 0.04 for FAP‐O‐VC‐MMAE; −0.52 ± 0.02 for FAP‐O‐GP‐MMAE), whereas the peptide‐based construct was substantially more hydrophilic (logD = −1.72 ± 0.02; Table 1). Radio‐HPLC analysis showed a single predominant peak for each [^68^Ga]Ga‐labeled conjugate after 2 h incubation in formulation solution, phosphate‐buffered saline (PBS), and mouse or human serum, indicating high short‐term in vitro stability (Figure 3A).

**TABLE 1:** Radiochemical characteristics and lipophilicity of the ^68^Ga‐Labeled SMDCs.

	[^68^Ga]Ga‐FAP‐O‐VC‐MMAE	[^68^Ga]Ga‐FAP‐O‐GP‐MMAE	[^68^Ga]Ga‐FAP‐P‐GP‐MMAE
Productivity	42.65 ± 6.95%	54.93 ± 3.91%	51.57 ± 3.08%
Radiochemical Purity	>99%	>99%	>99%
Molar Activity (GBq/µmol)	24.95 ± 6.79	31.47 ± 1.65	32.49 ± 6.03
Log*D*	−0.58 ± 0.04	−0.52 ± 0.02	−1.72 ± 0.02

For therapeutic studies, the corresponding [^177^Lu]Lu‐labeled conjugates were prepared from the same molecular scaffold. Radio‐HPLC and UV co‐elution confirmed the identity of the radiolabeled species and radiochemical purity exceeding 98% for both [^177^Lu]Lu‐FAP‐O‐VC‐MMAE and [^177^Lu]Lu‐FAP‐O‐GP‐MMAE (Figure 3B). Importantly, in vivo stability analysis in mouse blood at 24 h post‐injection revealed clear linker‐dependent differences: [^177^Lu]Lu‐FAP‐O‐VC‐MMAE remained 84.9% intact, whereas [^177^Lu]Lu‐FAP‐O‐GP‐MMAE retained 75.7% of the intact conjugate (Figure 3C). The intact fractions were quantified by radio‐HPLC peak integration. These results suggest that within this multifunctional SMDC architecture, the VC linker provides improved systemic stability relative to the GP motif.

To verify enzyme‐triggered payload release, FAP‐O‐VC‐MMAE was incubated with cathepsin B (CTSB). HPLC analysis revealed time‐dependent loss of the intact conjugate and the emergence of two product peaks. The later‐eluting peak was identified as MMAE by co‐elution with an authentic standard and high‐resolution mass spectrometry (m/z 718.5114 for [M+H]^+^; calc. 718.5119; 0.7 ppm) (Figure 3D). The earlier peak corresponded to the expected FAP‐O‐VC‐DOTA fragment (Figure S13). Collectively, these results confirm efficient CTSB‐mediated cleavage of the VC linker and demonstrate that linker chemistry can strongly influence both enzymatic payload release and systemic stability in this theranostic SMDC platform.

### 2.2 In Vitro Targeting and Cytotoxic Activity of FAP‐targeted SMDCs

**FIGURE 4:**
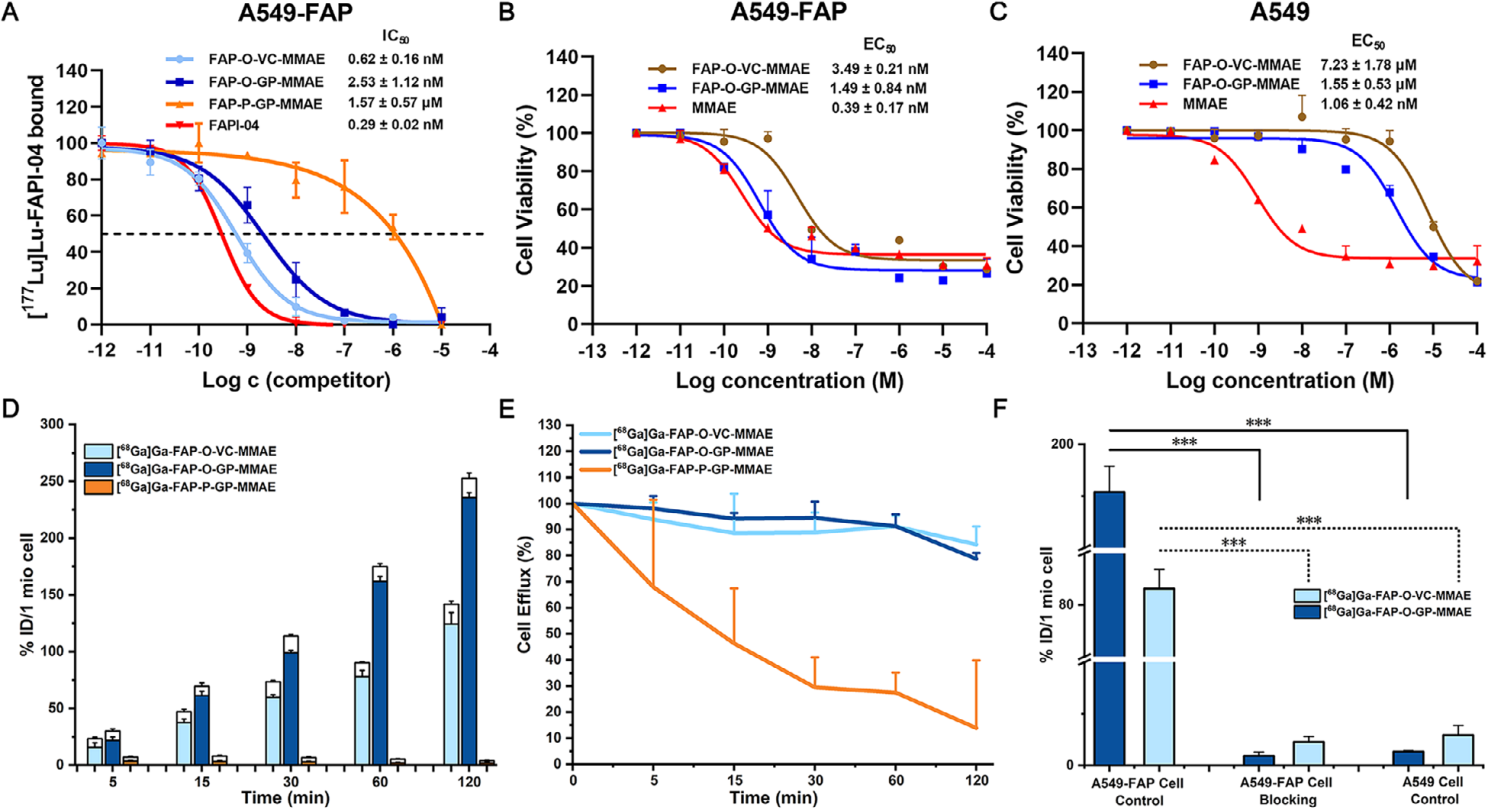
In vitro targeting specificity and cytotoxicity of FAP‐targeted SMDCs. (A) Competitive binding curves of SMDCs against [^177^Lu]Lu‐FAPI‐04 in FAP‐expressing cells. IC_50_ values were determined by nonlinear regression analysis. (B, C) Cytotoxicity of FAP‐O‐VC‐MMAE and FAP‐O‐GP‐MMAE against (B) FAP‐positive A549‐FAP cells and (C) FAP‐negative A549 cells. EC_50_ values were calculated by nonlinear regression analysis. (D) Cellular internalization kinetics of [^68^Ga]Ga‐labeled SMDCs in A549‐FAP cells. (E) Efflux profiles of internalized radioactivity following cellular internalization. (F) Validation of FAP‐mediated uptake using receptor blockade with excess FAPI‐04 and negligible uptake in FAP‐negative A549 cells. Data are presented as mean ± SD from technical replicates (n = 4). Statistical significance in panel F was assessed using one‐way ANOVA followed by Tukey's multiple comparisons test (***p < 0.001).

FAP binding was quantified by competition against [^177^Lu]Lu‐FAPI‐04 (Figure 4A). The FAP‐O scaffold tolerated installation of DOTA, linker, and MMAE with minimal affinity loss: IC_50_ 0.62 ± 0.16 nM (FAP‐O‐VC‐MMAE) and 2.53 ± 1.12 nM (FAP‐O‐GP‐MMAE), comparable to FAPI‐04 (0.29 ± 0.02 nM). In contrast, the FAP‐P‐GP‐MMAE construct exhibited a pronounced reduction in apparent affinity (IC_50_ 1.57 ± 0.57 µM), consistent with conjugation‐induced conformational penalties that can be especially detrimental for peptide‐based binding motifs in bulky multifunctional architectures. Functionally, this affinity gap translated into sharply different cellular behavior.

In CCK‐8 assays (Figure 4B,C), FAP‐O‐VC‐MMAE and FAP‐O‐GP‐MMAE were highly potent in FAP‐positive A549‐FAP cells (EC_50_ 3.49 ± 0.21 nM and 1.49 ± 0.84 nM, respectively), while being far less cytotoxic in FAP‐negative A549 cells (EC_50_ 7.23 ± 1.78 µM and 1.55 ± 0.53 µM). Thus, conjugation markedly reduced the non‐specific toxicity characteristic of free MMAE while preserving strong FAP‐dependent potency. Notably, the GP‐linked conjugate showed higher activity in both FAP‐positive and parental cells, hinting at a faster‐release phenotype that can be advantageous in vitro but may erode the safety margin in vivo.

Cellular internalization and efflux using [^68^Ga]Ga analogs further reinforced this interpretation (Figure 4D,E). Both [^68^Ga]Ga‐FAP‐O‐VC‐MMAE and [^68^Ga]Ga‐FAP‐O‐GP‐MMAE displayed rapid uptake into A549‐FAP cells with slow efflux (only ∼15–20% of internalized activity released at 120 min), whereas [^68^Ga]Ga‐FAP‐P‐GP‐MMAE showed negligible uptake and rapid clearance. Specificity was confirmed by near‐complete blockade (>99%) in the presence of excess FAPI‐04 and by low uptake in A549 controls (*p* < 0.001; Figure 4F). Collectively, these data identify FAP‐O as the more robust targeting vector for this theranostic SMDC format, in which chelation, radiometal loading, and hydrophobic payload installation impose substantial structural and physicochemical constraints.

### 2.3 PET Imaging Reveals Efficient Tumor Targeting and Linker‐Dependent Pharmacokinetics

**FIGURE 5:**
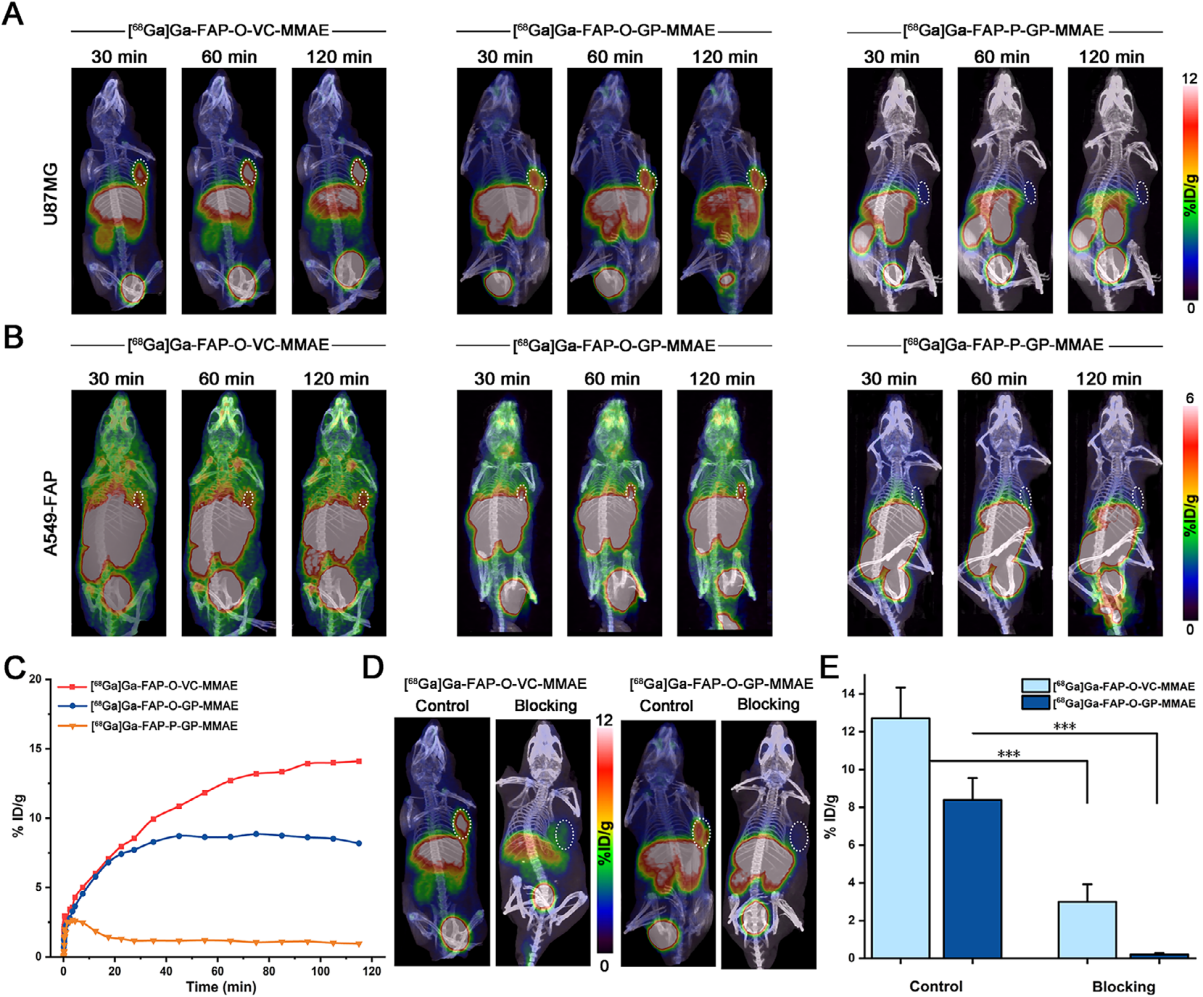
In vivo PET imaging and tumor targeting of FAP‐targeted SMDCs. Representative PET images of [^68^Ga]Ga‐labeled SMDCs in (A) U87MG and (B) A549‐FAP tumor‐bearing mice at 2 h post‐injection. (C) Tumor time–activity curves derived from dynamic PET scans in U87MG tumor‐bearing mice. (D) Representative PET images of U87MG tumor‐bearing mice acquired 1 h after injection of [^68^Ga]Ga‐labeled SMDCs with or without pre‐injection of excess FAPI‐04. (E) Quantification of tumor uptake demonstrating reduced tracer accumulation after receptor blocking. Data are presented as mean ± SD (n = 3). Statistical significance was assessed using a two‐tailed unpaired Student's t‐test (***p < 0.001). White circles indicate tumors.

Dynamic PET imaging up to 2 h post‐injection (Figure 5) demonstrated strong tumor targeting for the two FAP‐O constructs in both U87MG and A549‐FAP models, but with clearly higher accumulation in stroma‐rich U87MG xenografts than in the tumor cell–predominant A549‐FAP model. This observation aligns with the biological rationale for FAP targeting—leveraging CAF‐rich stroma as a drug reservoir—and emphasizes that stromal accessibility and abundance can be decisive for delivery efficiency and treatment impact [39, 40].

Time–activity curves in U87MG further exposed linker‐dependent kinetics (Figure 5C). [^68^Ga]Ga‐FAP‐O‐VC‐MMAE accumulated progressively throughout the scan, reaching 14.10% ID/g at 120 min, whereas [^68^Ga]Ga‐FAP‐O‐GP‐MMAE peaked lower (8.85% ID/g at 75 min) and then declined. [^68^Ga]Ga‐FAP‐P‐GP‐MMAE exhibited low, transient tumor uptake (peak 2.68% ID/g at 3.5 min). Receptor blocking with FAPI‐04 markedly reduced tumor uptake for both FAP‐O tracers (VC: 12.71 ± 1.63 to 3.00 ± 0.92% ID/g; GP: 8.38 ± 1.16 to 0.21 ± 0.08% ID/g, *p* < 0.001), confirming FAP‐mediated targeting (Figure 5D,E).

Biodistribution patterns also reflected the payload‐driven shift in physicochemical properties. The FAP‐O conjugates showed prominent hepatic uptake consistent with their moderate lipophilicity and hepatobiliary handling of hydrophobic scaffolds, whereas the more hydrophilic peptide‐based construct showed comparatively higher renal features on imaging. These PET‐resolved trade‐offs underscore the value of embedding an imaging reporter directly into the SMDC: biodistribution and exposure can be assessed non‐invasively and iteratively during optimization, rather than inferred from affinity or cell assays alone.

### 2.4 Biodistribution and Exposure Analysis Reveal Linker‐Dependent Pharmacokinetics

**FIGURE 6:**
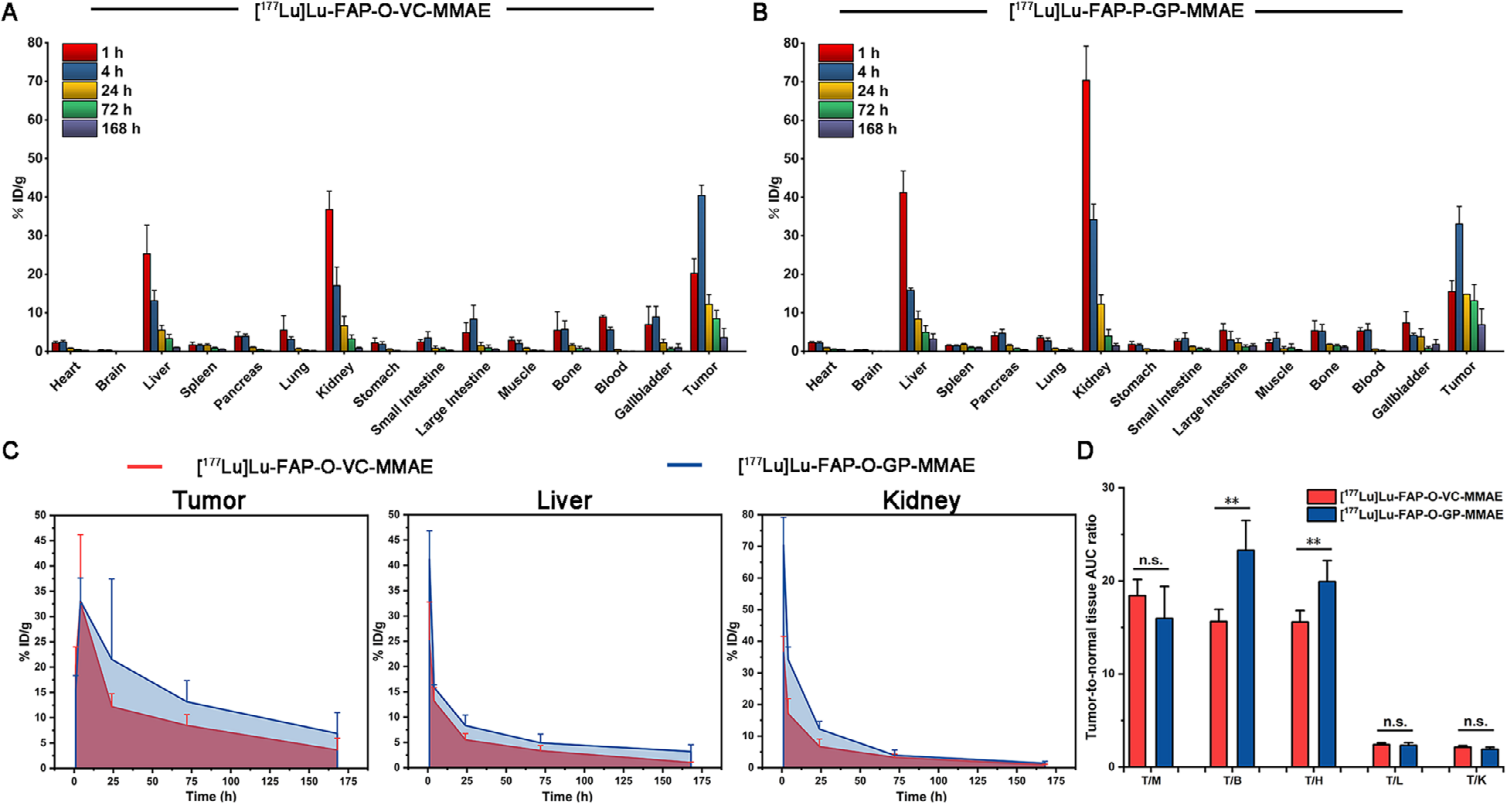
Biodistribution and pharmacokinetics of [^177^Lu]Lu‐labeled SMDCs in U87MG tumor‐bearing mice. (A, B) Time‐dependent biodistribution of (A) [^177^Lu]Lu‐FAP‐O‐VC‐MMAE and (B) [^177^Lu]Lu‐FAP‐O‐GP‐MMAE in major tissues. (C) Area under the curve (AUC) analysis of radioconjugate accumulation in tumors, liver, and kidneys. (D) Tumor‐to‐normal tissue AUC ratios. Tumor (T); Muscle (M); Blood (B); Heart (H); Liver (L); Kidney (K). Data are presented as mean ± SD (n = 3 per time point). Statistical significance was assessed using a two‐tailed unpaired Student's t‐test (**p < 0.01; n.s., not significant).

Ex vivo biodistribution of the therapeutic radionuclide conjugates in U87MG‐bearing mice (n = 3 per time point, Figure 6) showed that both [^177^Lu]Lu‐FAP‐O‐VC‐MMAE and [^177^Lu]Lu‐FAP‐O‐GP‐MMAE accumulated prominently in tumors, liver, and kidneys. Tissue radioactivity was quantified using representative samples collected from defined anatomical regions (e.g., quadriceps muscle and femoral bone). Tumor uptake peaked at 4 h for both agents (32.74 ± 13.44% ID/g for VC; 33.05 ± 4.55% ID/g for GP). However, early organ uptake differed substantially: the GP conjugate exhibited significantly higher 1 h uptake in kidney (70.38 ± 18.88 vs. 36.77 ± 4.80% ID/g) and liver (41.20 ± 5.63 vs. 25.28 ± 7.48% ID/g), followed by a more rapid decline.

Integrated exposure analysis (AUC, 1–168 h) revealed higher cumulative accumulation for [^177^Lu]Lu‐FAP‐O‐GP‐MMAE in tumor (2412.01 ± 256.6 vs. 1603.70 ± 110.7%ID/g·h), liver (1035.80 ± 63.4 vs. 666.58 ± 35.9%ID/g·h), and kidneys (1266.74 ± 60.7 vs. 755.19 ± 48.8%ID/g·h) (p < 0.01; Figure 6C). Tumor‐to‐blood and tumor‐to‐heart AUC ratios were also higher for the GP construct (Figure 6D), indicating improved tumor‐to‐background contrast in certain compartments. However, the increased hepatic and renal exposure may have important translational implications, especially for therapeutic applications and repeated dosing.

Taken together with the 24 h blood stability results (VC > GP), these findings indicate that linker chemistry strongly influences both systemic stability and organ‐level exposure. Such effects become particularly important in radiometal‐bearing SMDCs, where metabolic processing and clearance pathways may amplify the consequences of premature linker cleavage. Together, these pharmacokinetic and exposure differences suggest that linker chemistry may critically shape the therapeutic index of the platform, prompting further evaluation of how these linker‐dependent properties translate into antitumor efficacy and tolerability in vivo.

### 2.5 Combined Radionuclide–Chemotherapy Achieves Potent Tumor Suppression With Linker‐Governed Tolerability

**FIGURE 7:**
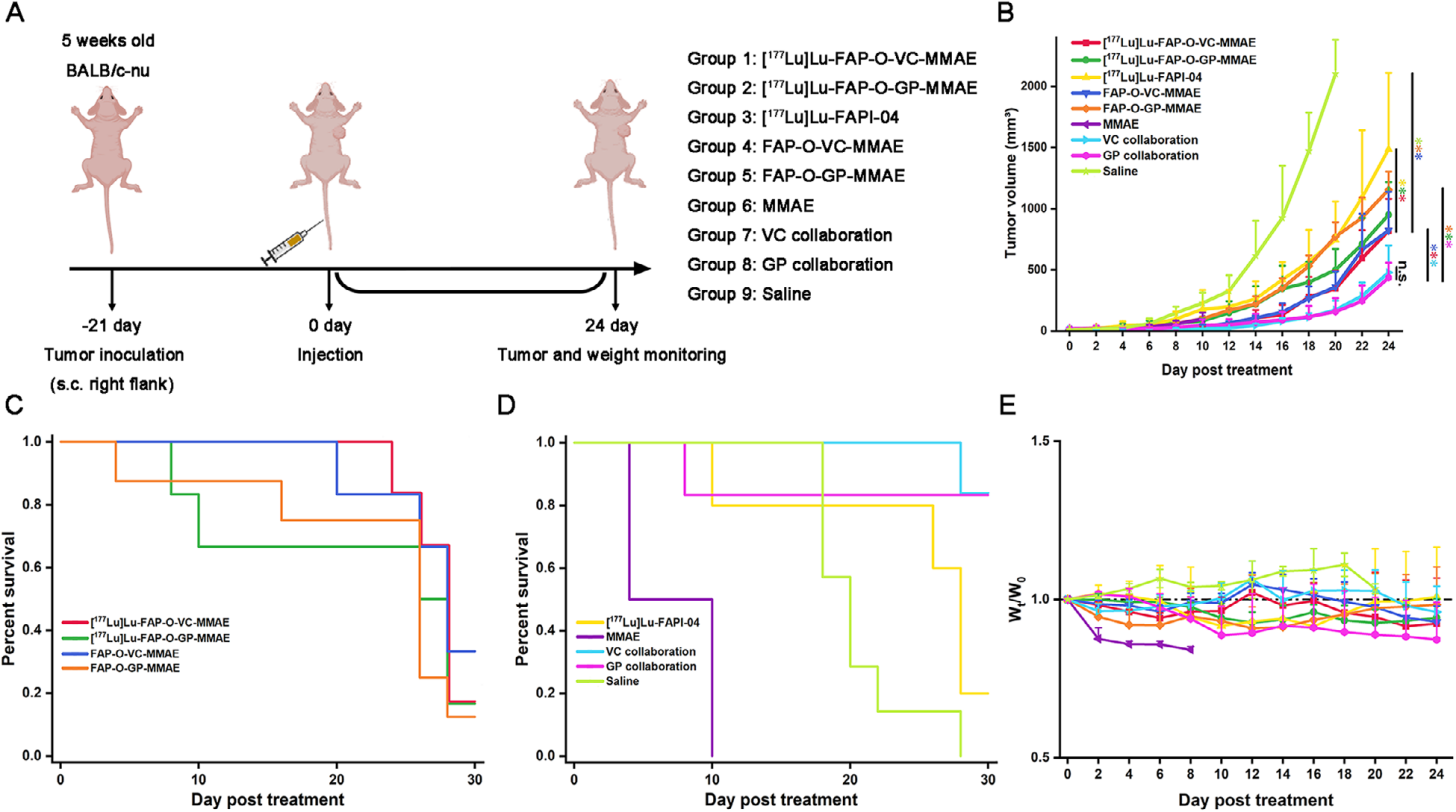
In vivo anti‐tumor efficacy of FAP‐targeted SMDC‐based therapies in U87MG tumor‐bearing mice. (A) Schematic illustration of the treatment schedule and group allocation. (B) Tumor growth curves of the indicated treatment groups over a 24‐day period. (C) Kaplan–Meier survival curves of monotherapy groups (Groups 1–2 and 4–5). (D) Kaplan–Meier survival curves of control and combination groups (Groups 3, 6–9). (E) Time‐dependent changes in body weight relative to the initial body weight. Data are presented as mean ± SD (n = 6 per group). Statistical analyses were performed using one‐way ANOVA followed by Dunnett's multiple comparisons test. Statistical significance was defined as ***p < 0.001; n.s., not significant. Combination groups correspond to co‐administration of [^177^Lu]Lu‐FAP‐O‐VC‐MMAE with FAP‐O‐VC‐MMAE (VC combination) or [^177^Lu]Lu‐FAP‐O‐GP‐MMAE with FAP‐O‐GP‐MMAE (GP combination).

Therapeutic evaluation in U87MG‐bearing mice (Figure 7) demonstrated that the integrated theranostic SMDC platform enables potent antitumor activity and provides mechanistic insights into linker‐dependent performance. All active regimens significantly inhibited tumor growth compared with the saline control (Group 9) (Figure 7A,B).

Importantly, targeted radiochemotherapy outperformed either treatment modality alone. Both [^177^Lu]Lu‐FAP‐O‐VC‐MMAE and [^177^Lu]Lu‐FAP‐O‐GP‐MMAE produced stronger tumor growth inhibition than [^177^Lu]Lu‐FAPI‐04 (Group 3), indicating that co‐delivery of MMAE through the same FAP‐targeting scaffold enhances efficacy beyond radionuclide therapy alone. This trend is consistent with the corresponding monotherapy datasets (Figure 7C). In contrast, free MMAE (Group 6) caused severe systemic toxicity requiring early termination, underscoring the necessity of targeted delivery for MMAE‐based therapy.

Crucially, combination therapy—pairing the therapeutic radionuclide conjugate with its corresponding non‐radioactive SMDC—produced the strongest antitumor responses. Tumor growth inhibition (TGI) reached 92.3–93.3% in the VC and GP combination groups (Groups 7–8), significantly exceeding all monotherapy regimens (Figure S17). Consistent with these results, Kaplan–Meier survival analysis further demonstrated the therapeutic advantage of the combined regimens. Survival outcomes of monotherapy groups are shown in Figure 7C, while comparisons among combination and control groups are presented in Figure 7D, both indicating prolonged survival in the combination groups relative to other treatments.

This enhanced efficacy is consistent with mechanistic synergy between β‐particle crossfire irradiation from ^177^Lu and microtubule‐disrupting cytotoxicity from MMAE, enabling simultaneous damage to stromal and adjacent tumor compartments.

Despite comparable antitumor efficacy between the two combination strategies, tolerability differed markedly between linker systems. VC‐linked treatments (Groups 1, 4, 7) maintained stable body weight, whereas GP‐linked regimens (Groups 2, 5, 8) induced significant weight loss (p < 0.05, Figure 7E), suggesting increased systemic toxicity. When interpreted alongside the biodistribution and blood stability results (Sections 2.1 and 2.4), these findings indicate that linker chemistry plays a decisive role in shaping the safety–efficacy balance of the platform. The GP linker—although previously reported as promising in certain FAP‐SMDC contexts [24]—appears more susceptible to premature payload release in the present multifunctional architecture. However, this conclusion is based on indirect evidence, and further studies such as metabolite profiling and direct quantification of released MMAE would be required to confirm the underlying mechanism.

This context dependence likely reflects emergent properties of the full molecular construct, including altered plasma protein interactions, hepatobiliary processing, and protease accessibility during liver‐associated trafficking. Notably, the relatively high hepatic uptake observed for the FAP‐O conjugates provides a potential amplification mechanism, whereby even modest increases in off‐target cleavage could translate into elevated systemic exposure and weight loss. Future studies incorporating quantitative analysis of circulating free MMAE and key catabolites, together with plasma and hepatic metabolite profiling, will help further clarify the mechanistic basis of GP‐associated toxicity in this system.

### 2.6 Implications for Imaging‐Guided Optimization of Radiochemotherapeutic SMDCs

Overall, the integrated results establish a clear structure–stability–efficacy relationship that cannot be inferred from binding affinity or in vitro potency alone. The FAP‐O targeting vector retained nanomolar affinity after conjugation and enabled robust cellular uptake together with strong PET‐defined tumor accumulation, whereas the peptide‐based ligand proved highly sensitive to conjugation and consistently underperformed across assays.

Linker chemistry, in contrast, primarily governed systemic stability and tolerability. The clinically validated VC linker supported efficient CTSB‐triggered MMAE release while maintaining higher in vivo integrity and improved tolerability. The GP linker, although associated with higher exposure and greater apparent in vitro potency, resulted in reduced tolerability in vivo.

Embedding PET functionality within the SMDC scaffold therefore provides a practical strategy for imaging‐guided optimization of radiochemotherapeutic SMDCs. This approach enables direct visualization of pharmacokinetics and tumor delivery in vivo, facilitating rapid identification of molecular designs that maximize therapeutic synergy while preserving an acceptable safety window [41, 42].

## 3 Conclusions

This study presents a rationally designed FAP‐targeted SMDC system as a theranostic strategy that enables coordinated evaluation of in vivo behavior and therapeutic performance. A unified molecular construct was generated by integrating the FAP‐O targeting ligand, enzymatically cleavable linkers (VC and GP), a DOTA chelator for radiometal complexation, and the cytotoxic payload MMAE to support tumor‐selective delivery and controlled payload release. The VC linker‐based conjugates exhibited favorable characteristics, including enhanced plasma stability, efficient tumor targeting, and an improved tolerability profile, whereas GP‐linked constructs showed evidence of premature payload release associated with increased systemic toxicity. Collectively, these findings identify FAP‐O‐VC‐MMAE as a promising lead theranostic radio‐chemotherapy construct and provide insights into linker‐dependent release behavior and its impact on the therapeutic index, thereby supporting the rational development of FAP‐targeted cancer theranostic agents.

## 4 Experimental Section

All experimental procedures, including chemical synthesis, radiochemistry, in vitro assays, and in vivo studies, are described in detail in the Supporting Information.

All animal experiments were conducted in accordance with institutional guidelines and relevant regulations, and were approved by the Institutional Animal Care and Use Committee (IACUC) of Southern Medical University (Approval No.: IACUC‐LAC‐20250211‐003).

## Conflicts of Interest

The authors declare no conflicts of interest.

## Supporting information




**Supporting File**: advs75150‐sup‐0001‐SuppMat.docx.

## Data Availability

The data that support the findings of this study are available from the corresponding author upon reasonable request.
