# Supplementary material for: A FAP‐Targeted SMDC Platform Enables Synergistic Radionuclide–Chemotherapy with PET‐Guided Evaluation
Source: Adv Sci (Weinh). 2026 Apr 7;13(36):e75150. doi: 10.1002/advs.75150 (PMC13317686; doi:10.1002/advs.75150)
Supplement: Supplementary file 1 — Supporting File: advs75150‐sup‐0001‐SuppMat.docx. [file ADVS-13-e75150-s001.docx]

**Supporting Information for**

**Original article**

**A FAP-targeted SMDC platform enables synergistic radionuclide–chemotherapy with PET-guided evaluation**

**Table of contents**

**[1. Materials and methods 3](#_Toc153)**

**[1.1 Chemistry 3](#_Toc3503)**

*[tert-butyl((S)-1-(((S)-1-((4-(hydroxymethyl)phenyl)amino)-1-oxo-5-ureidopentan-2-yl)amino)-3-methyl-1-oxobutan-2-yl)carbamate](#_Toc26769)* ***[(14)](#_Toc26769)*****[4](#_Toc26769)**

*[4-((S)-2-((S)-2-amino-3-methylbutanamido)-5-ureidopentanamido)benzyl ((S)-1-(((S)-1-(((3R,4S,5S)-1-((S)-2-((1R,2R)-3-(((1S,2R)-1-hydroxy-1-phenylpropan-2-yl)amino)-1-methoxy-2-methyl-3-oxopropyl)pyrrolidin-1-yl)-3-methoxy-5-methyl-1-oxoheptan-4-yl)(methyl)amino)-3-methyl-1-oxobutan-2-yl)amino)-3-methyl-1-oxobutan-2-yl)(methyl)carbamate](#_Toc14254)* ***[(1)](#_Toc14254)*****[4](#_Toc14254)**

*[(S)-4-((((9H-fluoren-9-yl)methoxy)carbonyl)amino)-5-(4-(3-((4-((2-((S)-2-cyano-4,4-difluoropyrrolidin-1-yl)-2-oxoethyl)carbamoyl)quinolin-6-yl)(methyl)amino)propyl)piperazin-1-yl)-5-oxopentanoic acid](#_Toc14457)* ***[(2)](#_Toc14457)*****[5](#_Toc14457)**

*[4-((S)-2-((S)-2-((S)-4-amino-5-(4-(3-((4-((2-((S)-2-cyano-4,4-difluoropyrrolidin-1-yl)-2-oxoethyl)carbamoyl)quinolin-6-yl)(methyl)amino)propyl)piperazin-1-yl)-5-oxopentanamido)-3-methylbutanamido)-5-ureidopentanamido)benzyl ((S)-1-(((S)-1-(((3R,4S,5S)-1-((S)-2-((1R,2R)-3-(((1S,2R)-1-hydroxy-1-phenylpropan-2-yl)amino)-1-methoxy-2-methyl-3-oxopropyl)pyrrolidin-1-yl)-3-methoxy-5-methyl-1-oxoheptan-4-yl)(methyl)amino)-3-methyl-1-oxobutan-2-yl)amino)-3-methyl-1-oxobutan-2-yl)(methyl)carbamate](#_Toc865)* ***[(3)](#_Toc865)*****[6](#_Toc865)**

*[tert-butyl(S)-(2-(2-((4-(hydroxymethyl)phenyl)carbamoyl)pyrrolidin-1-yl)-2-oxoethyl)carbamate](#_Toc13247)* ***[(18)](#_Toc13247)*****[6](#_Toc13247)**

*[4-((S)-1-glycylpyrrolidine-2-carboxamido)benzyl((2S)-1-(((2R)-1-(((3R,4S,5S)-1-(2-((1R,2R)-3-(((1S,2R)-1-hydroxy-1-phenylpropan-2-yl)amino)-1-methoxy-2-methyl-3-oxopropyl)pyrrolidin-1-yl)-3-methoxy-5-methyl-1-oxoheptan-4-yl)(methyl)amino)-3-methyl-1-oxobutan-2-yl)amino)-3-methyl-1-oxobutan-2-yl)(methyl)carbamate](#_Toc17562)****[(20)](#_Toc17562)*****[7](#_Toc17562)**

*[N2-(tert-butoxycarbonyl)-N5-(2-((2S)-2-((4-(((((2S)-1-(((2R)-1-(((3R,4S,5S)-1-(2-((1R,2R)-3-(((1S,2R)-1-hydroxy-1-phenylpropan-2-yl)amino)-1-methoxy-2-methyl-3-oxopropyl)pyrrolidin-1-yl)-3-methoxy-5-methyl-1-oxoheptan-4-yl)(methyl)amino)-3-methyl-1-oxobutan-2-yl)amino)-3-methyl-1-oxobutan-2-yl)(methyl)carbamoyl)oxy)methyl)phenyl)carbamoyl)pyrrolidin-1-yl)-2-oxoethyl)-L-glutamine](#_Toc27799)****[(5)](#_Toc27799)*****[7](#_Toc27799)**

*[4-((S)-1-(((S)-4-amino-5-(4-(3-((4-((2-((S)-2-cyano-4,4-difluoropyrrolidin-1-yl)-2-oxoethyl)carbamoyl)quinolin-6-yl)(methyl)amino)propyl)piperazin-1-yl)-5-oxopentanoyl)glycyl)pyrrolidine-2-carboxamido)benzyl ((2S)-1-(((2R)-1-(((3R,4S,5S)-1-(2-((1R,2R)-3-(((1S,2R)-1-hydroxy-1-phenylpropan-2-yl)amino)-1-methoxy-2-methyl-3-oxopropyl)pyrrolidin-1-yl)-3-methoxy-5-methyl-1-oxoheptan-4-yl)(methyl)amino)-3-methyl-1-oxobutan-2-yl)amino)-3-methyl-1-oxobutan-2-yl)(methyl)carbamate](#_Toc25505)****[(8)](#_Toc25505)*****[8](#_Toc25505)**

*[(12S,32S,5R,13R,16S,19S,22S)-19-(3-amino-3-oxopropyl)-95-(((2-((2S)-2-amino-5-((2-((2S)-2-((4-((5S,8R,11S,12R)-12-(2-(2-((1R,2R)-3-(((1S,2R)-1-hydroxy-1-phenylpropan-2-yl)amino)-1-methoxy-2-methyl-3-oxopropyl)pyrrolidin-1-yl)-2-oxoethyl)-5,8,11-triisopropyl-4,10-dimethyl-3,6,9-trioxo-2,13-dioxa-4,7,10-triazatetradecyl)phenyl)carbamoyl)pyrrolidin-1-yl)-2-oxoethyl)amino)-5-oxopentanamido)ethyl)thio)methyl)-16-benzyl-5-hexanamido-22-((R)-1-hydroxyethyl)-2,4,15,18,21,24-hexaoxo-7,11-dithia-14,17,20,23-tetraaza-1(1,2),3(2,1)-dipyrrolidina-9(1,3)-benzenacyclotetracosaphane-13-carboxylic acid](#_Toc30067)****[(9)](#_Toc30067)*****[9](#_Toc30067)**

**[1.2 Experimental Section 9](#_Toc20317)**

*[1.2.1 General](#_Toc30442)* [9](#_Toc30442)

*[1.2.2 Chemical and radiochemical synthesis](#_Toc30230)* [10](#_Toc30230)

*[1.2.3 Distribution coefficient](#_Toc5641)* [10](#_Toc5641)

*[1.2.4 Cell Culture and Animal Models](#_Toc8443)* [10](#_Toc8443)

*[1.2.5 Stability studies in vitro and in vivo](#_Toc13877)* [11](#_Toc13877)

*[1.2.6 In Vitro Enzymatic Cleavage Assay](#_Toc12551)* [11](#_Toc12551)

*[1.2.7 Cytotoxicity Assay](#_Toc15102)* [11](#_Toc15102)

*[1.2.8 In Vitro Cell Experiments](#_Toc28680)* [12](#_Toc28680)

*[1.2.9 Small Animal PET Imaging](#_Toc20876)* [12](#_Toc20876)

*[1.2.10 Biodistribution](#_Toc32459)* [12](#_Toc32459)

*[1.2.11 In Vivo Therapy](#_Toc18074)* [13](#_Toc18074)

*[1.2.12 Statistical Analysis](#_Toc29116)* [13](#_Toc29116)

**[1.3 Analytical Characterization of Compounds 14](#_Toc21780)**

**[2. Supporting Results 24](#_Toc30592)**

**[2.1 In Vitro Cathepsin B Cleavage Assay 24](#_Toc31442)**

**[2.2 Individual Tumor Growth Curves from the In Vivo Therapy Study 25](#_Toc330)**

**[2.3 Tumor growth inhibition (TGI) analysis of all treatment groups.. 2](#_Toc3082)6**

1. **Materials and methods**

*1.1 Chemistry*

**Scheme 1** Synthetic route used to generate precursor 1 and 2.


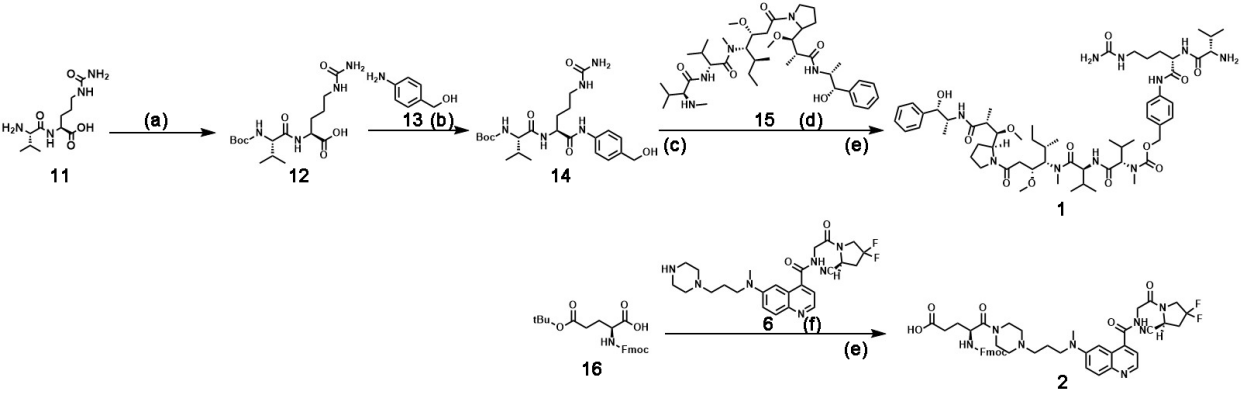


Reagents and conditions. (a) DIEA, DDM, rt, 12 h. (b) EEDQ, DCM, rt, 12 h. (c) DNPC, DIEA, DMF, rt, 2 h. (d) HOBT, DIEA, Pyridine, DMF, rt, 12 h. (e) TFA. (f) EDCI, HOOBT, NMM, DMF, rt, 3 h.

**Scheme 2** Synthetic route used to generate precursor 5.


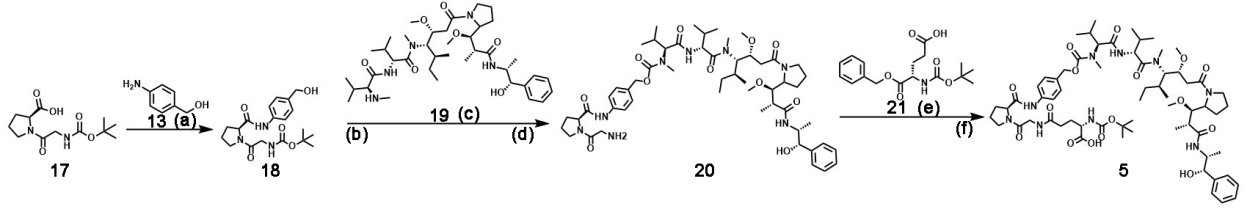


Reagents and conditions. (a) EEDQ, DCM, rt, 12 h. (b) DNPC, DIEA, DMF, rt, 2 h. (c) HOBT, DIEA, Pyridine, DMF, rt, 12 h. (d) TFA. (e) EDCI, HOBT, NMM, DMF, rt, 3 h. (f) Pd/C, H_2_, MeOH, rt, 4 h.

**Abbreviations**

| **DIEA** | N,N-diisopropylethylamine |
| --- | --- |
| **DDM** | 4,4'-Diaminodiphenylmethane |
| **EEDQ** | 2-Ethoxy-1-ethoxycarbonyl-1,2-dihydroquinoline |
| **DCM** | Dichloromethane |
| **DNPC** | Bis(4-nitrophenyl) carbonate |
| **DMF** | N,N-dimethylformamide |
| **HOBT** | 1-Hydroxybenzotriazole |
| **TFA** | trifluoroacetic acid |
| **EDCI** | 1-ethyl-3-(3-dimethylaminopropyl)carbodiimide |
| **HOOBt** | 1-hydroxy-7-azabenzotriazole |
| **NMM** | N-methylmorpholine |
| **PIP** | piperidine |
| **HATU** | O-(7-Azabenzotriazol-1-yl)-N,N,N',N'-tetramethyluronium hexafluorophosphate |
| **TSTU** | N,N,N′,N′-tetramethyl-O-(N-succinimidyl)uronium tetrafluoroborate |

*tert-butyl((S)-1-(((S)-1-((4-(hydroxymethyl)phenyl)amino)-1-oxo-5-ureidopentan-2-yl)amino)-3-methyl-1-oxobutan-2-yl)carbamate* ***(14)***

Dissolve Compound 2 (412 mg, 1.1 mmol) in dichloromethane (20 mL). To this solution, add Compound 3 (407 mg, 3.3 mmol) and EEDQ (815 mg, 3.3 mmol). Stir the reaction mixture at room temperature overnight. After confirming reaction completion by LC-MS, concentrate the mixture under reduced pressure to afford the crude product. Purify the crude material by reverse-phase chromatography to obtain Compound 4 (500 mg). MS (ESI-TOF) (*m/z*): calcd for C_23_H_37_N_5_O_6_ [M + H]^+^, 480.28, found, 480.28.

*4-((S)-2-((S)-2-amino-3-methylbutanamido)-5-ureidopentanamido)benzyl ((S)-1-(((S)-1-(((3R,4S,5S)-1-((S)-2-((1R,2R)-3-(((1S,2R)-1-hydroxy-1-phenylpropan-2-yl)amino)-1-methoxy-2-methyl-3-oxopropyl)pyrrolidin-1-yl)-3-methoxy-5-methyl-1-oxoheptan-4-yl)(methyl)amino)-3-methyl-1-oxobutan-2-yl)amino)-3-methyl-1-oxobutan-2-yl)(methyl)carbamate* ***(1)***

Compound 4 (500 mg, 0.96 mmol) and DNCP (1.2 g, 3.97 mmol) were dissolved in DMF (5 mL), followed by addition of DIEA (655 μL, 3.97 mmol); the reaction was stirred at room temperature for 2 hours. After LC-MS confirmed completion, the mixture was concentrated and purified by reverse-phase chromatography to afford Boc-Val-Cit-PAB-PNP (200 mg). This intermediate was dissolved in DMF (5 mL), then treated with Compound 5 (100 mg, 0.14 mmol), HOBt (19 mg, 0.14 mmol), DIEA (437 μL, 2.64 mmol), and pyridine (1.5 mL), and stirred at room temperature overnight. LC-MS indicated full conversion; the mixture was concentrated, and the residue was treated with TFA (3 mL) at room temperature for 3 minutes. The product was precipitated by addition to ice‑cold diethyl ether (30 mL), isolated by centrifugation, and dried, then purified by reverse‑phase preparative chromatography to give Compound 6 (60 mg). MS (ESI-TOF) (*m/z*): calcd for C_58_H_94_N_10_O_12_ [M + H]^+^, 1123.71, found, 1124.09.

*(S)-4-((((9H-fluoren-9-yl)methoxy)carbonyl)amino)-5-(4-(3-((4-((2-((S)-2-cyano-4,4-difluoropyrrolidin-1-yl)-2-oxoethyl)carbamoyl)quinolin-6-yl)(methyl)amino)propyl)piperazin-1-yl)-5-oxopentanoic acid* ***(2)***

Compound 7 (3.4 mg, 0.008 mmol) was dissolved in anhydrous DMF (3 mL). To this stirred solution at room temperature were added HOOBT (12.8 mg, 0.071 mmol), EDCI hydrochloride (13.6 mg, 0.071 mmol), and N-methylmorpholine (20 μL, 0.18 mmol). The reaction mixture was stirred for 5 minutes to allow for in-situ formation of the active ester. Subsequently, Compound 8 (4 mg, 0.008 mmol) was added in one portion. The resulting mixture was stirred at room temperature for 3 hours, at which point LC-MS analysis indicated complete consumption of the starting material. The reaction mixture was concentrated under reduced pressure to remove DMF. The crude intermediate was then treated with neat TFA (3 mL) at room temperature for 3 minutes to achieve simultaneous deprotection. The TFA solution was then added dropwise into vigorously stirred ice-cold diethyl ether (30 mL) to precipitate the product. The resulting solid was collected by centrifugation, washed with cold diethyl ether, and dried under a stream of nitrogen. Final purification by reverse-phase preparative HPLC (C18 column, water/acetonitrile gradient with 0.1% TFA modifier) yielded Compound 9 as a white solid. MS (ESI-TOF) (*m/z*): calcd for C_45_H_48_F_2_N_8_O_7_ [M + H]^+^, 851.37, found, 851.48.

*4-((S)-2-((S)-2-((S)-4-amino-5-(4-(3-((4-((2-((S)-2-cyano-4,4-difluoropyrrolidin-1-yl)-2-oxoethyl)carbamoyl)quinolin-6-yl)(methyl)amino)propyl)piperazin-1-yl)-5-oxopentanamido)-3-methylbutanamido)-5-ureidopentanamido)benzyl ((S)-1-(((S)-1-(((3R,4S,5S)-1-((S)-2-((1R,2R)-3-(((1S,2R)-1-hydroxy-1-phenylpropan-2-yl)amino)-1-methoxy-2-methyl-3-oxopropyl)pyrrolidin-1-yl)-3-methoxy-5-methyl-1-oxoheptan-4-yl)(methyl)amino)-3-methyl-1-oxobutan-2-yl)amino)-3-methyl-1-oxobutan-2-yl)(methyl)carbamate* ***(3)***

Compound 6 (60 mg, 0.054 mmol) was dissolved in anhydrous DMF (3 mL). HOOBT (12.8 mg, 0.071 mmol), EDCI (13.6 mg, 0.071 mmol), and NMM (20 μL, 0.18 mmol) were added sequentially at room temperature. The mixture was stirred for 5 minutes, followed by the addition of Compound 9 (46 mg, 0.054 mmol). The reaction was stirred at room temperature for 3 hours. After LC-MS confirmed complete consumption of starting materials, the reaction mixture was concentrated under reduced pressure to remove DMF. The crude intermediate was then treated with 20% piperidine in DMF (3 mL) at room temperature for 20 minutes to cleave a protecting group (e.g., Fmoc or similar base-labile group). The mixture was concentrated, and the residue was purified by reverse-phase preparative HPLC to afford Compound 10. MS (ESI-TOF) (*m/z*): calcd for C_88_H_130_F_2_N_18_O_16_ [M + 2H]^2+^, 867.50, found, 867.61.

*tert-butyl(S)-(2-(2-((4-(hydroxymethyl)phenyl)carbamoyl)pyrrolidin-1-yl)-2-oxoethyl)carbamate****(18)***

Compound 12 (300 mg, 1.1 mmol) was dissolved in dichloromethane (20 mL). To this solution were added Compound 3 (407 mg, 3.3 mmol) and EEDQ (815 mg, 3.3 mmol). The reaction mixture was stirred at room temperature overnight. After LC-MS analysis confirmed completion, the mixture was concentrated under reduced pressure to dryness. The crude product was purified by reverse-phase chromatography to yield 500 mg of Compound 13. MS (ESI-TOF) (*m/z*): calcd for C_19_H_27_N_3_O_6_ [M + H]^+^, 378.20, found, 378.68.

*4-((S)-1-glycylpyrrolidine-2-carboxamido)benzyl((2S)-1-(((2R)-1-(((3R,4S,5S)-1-(2-((1R,2R)-3-(((1S,2R)-1-hydroxy-1-phenylpropan-2-yl)amino)-1-methoxy-2-methyl-3-oxopropyl)pyrrolidin-1-yl)-3-methoxy-5-methyl-1-oxoheptan-4-yl)(methyl)amino)-3-methyl-1-oxobutan-2-yl)amino)-3-methyl-1-oxobutan-2-yl)(methyl)carbamate****(20)***

The synthesis of Compound 14 was achieved through a sequential two-step procedure. First, Compound 13 (500 mg, 1.32 mmol) and bis(4-nitrophenyl) carbonate (1.2 g, 3.97 mmol) were dissolved in DMF (5 mL), followed by the addition of DIEA (655 µL, 3.97 mmol). The reaction mixture was stirred at room temperature for 2 hours. After confirming complete conversion by LC-MS, the mixture was concentrated under reduced pressure and purified by reverse-phase preparative chromatography to yield the activated intermediate Boc-Gly-Pro-PAB-PNP (200 mg). This intermediate was then dissolved in DMF (5 mL), and Compound 5 (100 mg, 0.14 mmol), HOBt (19 mg, 0.14 mmol), DIEA (437 µL, 2.64 mmol), and pyridine (1.5 mL) were added. The reaction mixture was stirred at room temperature overnight. Upon LC-MS confirmation of completion, the mixture was concentrated under reduced pressure to dryness. The residue was treated with TFA (3 mL) at room temperature for 3 minutes, followed by precipitation into ice-cold diethyl ether (30 mL). The resulting solid was collected by centrifugation, dried, and finally purified by reverse-phase preparative chromatography to afford 60 mg of Compound 14. MS (ESI-TOF) (*m/z*): calcd for C_54_H_84_N_8_O_11_ [M + H]^+^, 1021.63, found, 1021.88.

*N2-(tert-butoxycarbonyl)-N5-(2-((2S)-2-((4-(((((2S)-1-(((2R)-1-(((3R,4S,5S)-1-(2-((1R,2R)-3-(((1S,2R)-1-hydroxy-1-phenylpropan-2-yl)amino)-1-methoxy-2-methyl-3-oxopropyl)pyrrolidin-1-yl)-3-methoxy-5-methyl-1-oxoheptan-4-yl)(methyl)amino)-3-methyl-1-oxobutan-2-yl)amino)-3-methyl-1-oxobutan-2-yl)(methyl)carbamoyl)oxy)methyl)phenyl)carbamoyl)pyrrolidin-1-yl)-2-oxoethyl)-L-glutamine****(5)***

Compound 14 (20 mg, 0.059 mmol) was dissolved in DMF (3 mL). To this solution at 0°C were added HOBt (10 mg, 0.071 mmol), EDC (14 mg, 0.071 mmol), and NMM (20 µL, 0.18 mmol). The mixture was stirred for 5 minutes, followed by addition of Compound 15 (60 mg, 0.059 mmol). The reaction mixture was allowed to warm to room temperature and stirred for 3 hours. After LC-MS confirmed complete conversion, the mixture was concentrated under reduced pressure. The residue was dissolved in methanol (3 mL), and palladium on carbon (8 mg) was added. Hydrogenation was conducted under a hydrogen atmosphere at room temperature for 4 hours. After confirming completion by LC-MS, the catalyst was removed by filtration and the filtrate was concentrated under reduced pressure. Purification by reverse-phase preparative chromatography afforded Compound 16. MS (ESI-TOF) (*m/z*): calcd for C_64_H_99_N_9_O_16_ [M + H]^+^, 1250.73, found, 1251.21.

*4-((S)-1-(((S)-4-amino-5-(4-(3-((4-((2-((S)-2-cyano-4,4-difluoropyrrolidin-1-yl)-2-oxoethyl)carbamoyl)quinolin-6-yl)(methyl)amino)propyl)piperazin-1-yl)-5-oxopentanoyl)glycyl)pyrrolidine-2-carboxamido)benzyl ((2S)-1-(((2R)-1-(((3R,4S,5S)-1-(2-((1R,2R)-3-(((1S,2R)-1-hydroxy-1-phenylpropan-2-yl)amino)-1-methoxy-2-methyl-3-oxopropyl)pyrrolidin-1-yl)-3-methoxy-5-methyl-1-oxoheptan-4-yl)(methyl)amino)-3-methyl-1-oxobutan-2-yl)amino)-3-methyl-1-oxobutan-2-yl)(methyl)carbamate****(8)***

Compound 16 (10 mg, 0.008 mmol) was dissolved in anhydrous DMF (2 mL). To this solution were added HATU (3.6 mg, 0.0095 mmol) and DIEA (4 µL, 0.024 mmol) at room temperature, and the mixture was stirred for 30 minutes to allow for in-situ activation of the carboxyl group. Subsequently, Compound 8 (9 mg, 0.009 mmol) was added in one portion. The reaction mixture was stirred at room temperature for 3 hours. After confirming complete conversion by LC-MS, the mixture was concentrated under reduced pressure to remove DMF. The resulting residue was treated with neat TFA (2 mL) at room temperature for 3 minutes to achieve deprotection. The reaction mixture was then added dropwise into vigorously stirred ice-cold diethyl ether (20 mL) to precipitate the product. The resulting solid was collected by centrifugation, washed with cold diethyl ether, and dried under a stream of nitrogen. The crude product was purified by reverse-phase chromatography to afford 4 mg of Compound 17. MS (ESI-TOF) (*m/z*): calcd for C_84_H_119_F_2_N_16_O_15_ [M + 3H]^3+^, 544.31, found, 544.50.

*(12S,32S,5R,13R,16S,19S,22S)-19-(3-amino-3-oxopropyl)-95-(((2-((2S)-2-amino-5-((2-((2S)-2-((4-((5S,8R,11S,12R)-12-(2-(2-((1R,2R)-3-(((1S,2R)-1-hydroxy-1-phenylpropan-2-yl)amino)-1-methoxy-2-methyl-3-oxopropyl)pyrrolidin-1-yl)-2-oxoethyl)-5,8,11-triisopropyl-4,10-dimethyl-3,6,9-trioxo-2,13-dioxa-4,7,10-triazatetradecyl)phenyl)carbamoyl)pyrrolidin-1-yl)-2-oxoethyl)amino)-5-oxopentanamido)ethyl)thio)methyl)-16-benzyl-5-hexanamido-22-((R)-1-hydroxyethyl)-2,4,15,18,21,24-hexaoxo-7,11-dithia-14,17,20,23-tetraaza-1(1,2),3(2,1)-dipyrrolidina-9(1,3)-benzenacyclotetracosaphane-13-carboxylic acid****(9)***

Compound 16 (10 mg, 0.008 mmol) was dissolved in DMF (2 mL). TSTU (3 mg, 0.008 mmol) and DIEA (4 µL, 0.024 mmol) were added, and the mixture was stirred at room temperature for 30 minutes. Compound 18 (9 mg, 0.008 mmol) was then added, and stirring was continued at room temperature for 3 hours. After LC-MS analysis confirmed complete conversion, the reaction mixture was concentrated under reduced pressure. The residue was treated with TFA (2 mL) at room temperature for 3 minutes, followed by precipitation into ice-cold diethyl ether (20 mL). The resulting solid was collected by centrifugation and dried. The crude product was purified by reverse-phase chromatography to afford 6 mg of Compound 19. MS (ESI-TOF) (*m/z*): calcd for C_110_H_182_N_18_O_24_S_3_ [M + 2H]^2+^, 1108.06, found, 1108.35.

*1.2 Experimental Section*

*1.2.1 General*

All reagents used were purchased from commercially available companies and used without further purification unless otherwise indicated. Radiolabeling efficiency and radiochemical purity were tested using radio high performance liquid chromatography (HPLC). Imaging was performed for animals using Inveon small-animal PET/CT scanner (Siemens, Erlangen, Germany). ^68^GaCl_3_ was eluted from a Ge^68^/Ga^68^ generator (Itemba, South Africa). Carrier-free lutetium-177 (^177^LuCl_3_, 0.04 M HCl) was obtained from Isotopia Molecular Imaging Ltd. (Israel). Sep-Pak C18 cartridges (Waters) were purchased from Waters Associates. Radioactivity was measured using a γ-counter (CAPRAC-R, Capintec, Inc., Ramsey, NI). The other reagent supplier information and instrument details are provided in the supplementary data.

*1.2.2 Chemical and radiochemical synthesis*

The FAP-targeting SMDCs (FAP-O-VC-MMAE, FAP-O-GP-MMAE, and FAP-P-GP-MMAE) were synthesized via solid-phase methodology, achieving chemical purities exceeding 96% (synthetic routes detailed in Figure S1−S3, Supporting Information). Structural confirmation of key intermediates and final compounds was obtained through mass spectrometric analysis. For ^68^Ga-radiolabeling, a generator-eluted solution (2.0 mL, 481–518 MBq) was reacted with 13–14 nmol of the respective DOTA-conjugated precursor in 2.5 M HEPES buffer at 100 °C for 10 min. The crude product was diluted with 5.0 mL water and purified using a C18 solid-phase extraction cartridge. After washing with ethanol/water (1:1, *v*/*v*), the purified radiotracer was formulated in physiological saline with ethanol content maintained below 10%. For ^177^Lu-labeling, ^177^LuCl_3_ (120 MBq) was buffered with 0.16 mL sodium acetate (0.4 M, pH 5.2) and reacted with the DOTA-conjugated precursor (5 nmol) in the presence of gentisic acid (2.0 mg) at 95 °C for 20 min. Radiochemical purity was determined by radio-HPLC and exceeded 98% for all radiolabeled compounds[1].

*1.2.3 Distribution coefficient*

The partition coefficient (Log*D*) was determined using the shake-flask method according to established procedures[2]. Briefly, approximately 0.9 MBq of each radioligand was added to a mixture of 5.0 mL phosphate-buffered saline (PBS, pH 7.4) and 5.0 mL *n*-octanol. The biphasic system was vigorously vortexed for 5 min at room temperature followed by centrifugation at 4000 rpm for 3 min. Aliquots (*n* = 3) from each phase were collected and measured using a γ-counter (CAPRAC-R, Capintec, Inc., Ramsey, NJ, USA).

*1.2.4 Cell Culture and Animal Models*

A549-FAP cells (stably transfected), U87MG cells and A549 cells were cultured in DMEM (Gibco) supplemented with 10% FBS (Gibco) and 1% penicillin/streptomycin (Gibco)[3]. All animal experiments were approved by the Institutional Animal Care and Use Committee (IACUC) of Southern Medical University (Approval No.: IACUC-LAC-20250211-003). Male BALB/c-Nude mice (5–6 weeks old) were subcutaneously inoculated with U87MG or A549-FAP cells under right flank.

*1.2.5 Stability studies in vitro and in vivo*

[^68^Ga]Ga-FAP-O-VC-MMAE, [^68^Ga]Ga-FAP-O-GP-MMAE and [^68^Ga]Ga-FAP-P-GP-MMAE (18.5 MBq, 50 µL) were added to PBS (200 μL), mouse serum (MS, 200 μL) and human serum (HS, 200 μL). Respectively, the integrity of the radioligand was verified by radio-HPLC after the mixtures were incubated at 37 °C for 2 h. For *in vivo* stability study, the radiochemical products (18.5 MBq) were assessed in BALB/c-Nude mice by intravenous injection. 400 µL of blood was collected from the orbital venous plexus at 24 h post injection. Later, the same acetonitrile volume was introduced into the blood, and the mixture was centrifuged (10000 rpm, 5 min). The supernatant (100 µL) was collected for HPLC analysis, the fractions of the eluted sample were collected manually into a tube every 0.5 min for 15 min and measured with a γ-counter.

*1.2.6 In Vitro Enzymatic Cleavage Assay*

The enzymatic cleavage of the SMDC was investigated using cathepsin B (CTSB). The reaction buffer (pH 5.5) was prepared by mixing 0.4 M sodium acetate solution, a solution containing 30 mmol/L **d**ithio**t**hrei**t**ol and 15 mmol/L ethylenediaminetetraacetic acid, and ultrapure water. Both the FAP-O-VC-MMAE and MMAE reference standard were dissolved in this buffer. CTSB was reconstituted in the buffer to a final concentration of 120 nmol/L and activated for 15 min at room temperature. Subsequently, the activated CTSB solution (100 μL) was mixed with the solution of FAP-O-VC-MMAE (100 μL, 70.74 nmol), resulting in a final reaction volume of 200 μL with final concentrations of 60 nmol/L for CTSB and 353.7 μmol/L for the SMDC. The mixture was incubated at 37 °C for 4 h. The enzymatic reaction was quenched by transferring a 100 μL aliquot of the incubation mixture into a solution of acetonitrile/water (95:5, *v*/*v*). The samples were then analyzed by reversed-phase HPLC using a C18 column and a gradient of 5% to 95% acetonitrile in water (containing 0.1% TFA) over 30 min. Elution was monitored by a photodiode array detector at 254 nm. Fractions corresponding to the observed peaks were manually collected, lyophilized, and subjected to high-resolution mass spectrometry for structural identification. As controls, solutions of the intact FAP-O-VC-MMAE (100 μL, 23.58 nmol in buffer) and MMAE (100 μL from a 0.5 mg/mL stock in buffer) were separately analyzed by HPLC under identical conditions to establish their respective chromatographic profiles.

*1.2.7 Cytotoxicity Assay*

The cytotoxic activities of FAP-O-VC-MMAE, FAP-O-GP-MMAE, and free MMAE were evaluated *in vitro* using a Cell Counting Kit-8 (CCK-8) assay. Briefly, A549-FAP (FAP-positive) and A549 (FAP-negative) cells were seeded into 96-well plates at an appropriate density and allowed to adhere overnight. The cells were then treated with the respective compounds, which were serially diluted in the culture medium across a concentration range from 10^-4^ to 10^-12^ mol/L. Each concentration was tested in quadruplicate. Following 48 hours of incubation at 37 °C in a 5% CO₂ atmosphere, 10 μL of CCK-8 solution was added to each well, and the plates were further incubated for 1−4 h under light-protected conditions. The absorbance of each well was subsequently measured at a wavelength of 450 nm using a microplate reader. The half-maximal effective concentration (EC_50_) values were calculated from the dose-response curves using non-linear regression analysis in GraphPad Prism (version 9.5.0).

*1.2.8 In Vitro Cell Experiments*

As previously described, competitive binding assays were performed as follows: cells were incubated with [^177^Lu]Lu-FAPI-04 (37 KBq/mL) in the presence of increasing concentrations of unlabeled precursor (10^−5^–10^−11^ M) at 37 °C for 60 min[4]. Data were analyzed using GraphPad Prism (version 9.5.0) to calculate IC_50_ values. A549-FAP and A549 cells were seeded into 12-well plates and cultivated overnight. For the internalization studies, A549-FAP cells were incubated with radioligands (37 KBq/mL) for 5, 15, 30, 60 and 120 min at 37 °C. At the corresponding time points, the cells were washed with PBS and incubated for 10 min with 1 mL of glycine hydrochloride solution (1 M, pH 2.2) to detach the radioactivity bound to the cell surface. Afterward, the cells were then washed with PBS and lysed with NaOH (1 M, 2% SDS). Radioactivity was measured using a γ-counter. A549-FAP and A549 cells were incubated in [^68^Ga]Ga-FAP-O-VC-MMAE, [^68^Ga]Ga-FAP-O-GP-MMAE and [^68^Ga]Ga-FAP-P-GP-MMAE, with or without the competitor FAPI-04 (1 μM).

*1.2.9 Small Animal PET Imaging*

For *in vivo* PET imaging, U87MG tumor-bearing mice (*n* = 3 per group) were intravenously administered with ^68^Ga-labeled radioligands (11.10–12.95 MBq). Dynamic PET acquisition was performed continuously for 60 min post-injection using a Siemens scanner (Erlangen, Germany), followed by sequential static scans (10 min duration) at 60, 120, and 180 min. Specificity of uptake was assessed in blocking groups via co-injection with excess FAPI-04. Image analysis was performed using Inveon Research Workplace 4.1 software (Siemens), with regions of interest (ROIs) manually delineated on attenuation-corrected coronal whole-body images for quantification of radioactivity in major organs.

*1.2.10 Biodistribution*

In the biodistribution assessment, BALB/c nude mice received intravenous injections of ^177^Lu-labeled radioligands (1.85−3.00 MBq) and were sacrificed at designated intervals (1, 4, 24, 72, and 168 h). To evaluate targeting specificity, U87MG tumor-bearing mice (*n* = 3 per group) were administered [^177^Lu]Lu-FAP-O-VC-MMAE, [^177^Lu]Lu-FAP-O-GP-MMAE, or [^177^Lu]Lu-FAP-P-GP-MMAE. Following euthanasia, major organs and tissues (including heart, liver, lungs, kidneys, spleen, pancreas, stomach, gallbladder, small and large intestines, muscle, tumor, and brain) were immediately harvested and weighed. Muscle samples were collected from the quadriceps, and bone samples were obtained from the femur to ensure consistent anatomical sampling. Radioactivity quantification was performed using a γ-counter, with results normalized as percentage injected dose per gram of tissue (%ID/g).

*1.2.11 In Vivo Therapy*

U87MG tumor-bearing BALB/c nude mice were randomly divided into nine experimental groups (*n* = 6) using a random allocation procedure when tumor volumes reached approximately 15 mm^3^. The groups received single intravenous injections via tail vein as follows: Group 1: [^177^Lu]Lu-FAP-O-VC-MMAE (150 μL, 18.5 MBq), Group 2: [^177^Lu]Lu-FAP-O-GP-MMAE (150 μL, 18.5 MBq), Group 3: [^177^Lu]Lu-FAPI-04 (150 μL, 18.5 MBq), Group 4: FAP-O-VC-MMAE (150 μL, 50 μg), Group 5: FAP-O-GP-MMAE (150 μL, 50 μg), Group 6: MMAE (150 μL, 50 μg), Group 7: [^177^Lu]Lu-FAP-O-VC-MMAE (150 μL, 18.5 MBq) followed 8 hours later by FAP-O-VC-MMAE (150 μL, 50 μg), Group 8: [^177^Lu]Lu-FAP-O-GP-MMAE (150 μL, 18.5 MBq) followed 8 hours later by FAP-O-GP-MMAE (150 μL, 50 μg), Group 9: Saline (150 μL). All injections were administered via tail vein at a fixed volume of 150 μL per dose. The chemical amounts of radiolabeling precursors were 1 μg for FAP-O-VC-MMAE and FAP-O-GP-MMAE, and 0.5 μg for FAPI-04, respectively. Tumor volume and body weight were monitored every 2 days for 24 days. Animals were euthanized when tumor volume exceeded 1500 mm^3^ or body weight loss exceeded 20%, at which point the animal was humanely euthanized. Values were expressed as mean ± SD.

*1.2.12 Statistical Analysis*

Data are presented as mean ± standard deviation (SD). Sample sizes (n) for each experiment are specified in the corresponding figure legends. No data points were excluded from the analyses. Statistical analyses were performed using GraphPad Prism (version 9.5.0, GraphPad Software, San Diego, CA, USA), SPSS (version 27.0, IBM Corp., Armonk, NY, USA), and OriginPro (version 2024, OriginLab Corp., Northampton, MA, USA). Comparisons between two groups were evaluated using a two-tailed unpaired Student’s t-test. For comparisons among multiple groups, one-way analysis of variance (ANOVA) followed by Tukey’s or Dunnett’s multiple comparisons test was applied, as specified in the corresponding figure legends. Dose–response curves for cytotoxicity and competitive binding assays were analyzed by nonlinear regression to determine EC_50_ or IC_50_ values. Kaplan–Meier survival curves were generated using OriginPro 2024 and analyzed using the log-rank (Mantel–Cox) test. All statistical tests were two-sided. Differences were considered statistically significant at *P* < 0.05. Where applicable, data distribution was assumed to be approximately normal and variance was considered similar between groups. No formal test for normality was performed.

*1.3 Analytical Characterization of Compounds*

Analytical HPLC was performed on an LC-20AD system (Shimadzu, Japan) equipped with a DAD-UV detector and a B-FC-3200 high-energy PMT detector (Bioscan Inc., USA). Separations were conducted using a COSMOSIL 5C18-MS-Ⅱ analytical column (4.6 mm × 250 mm) at a flow rate of 1.0 mL/min, with detection at 220 nm via a Prominence SPD-M20A PDA detector (Shimadzu, Japan). For FAP-O-VC-MMAE and FAP-O-GP-MMAE, a linear gradient was applied from 67% solvent A (0.1% TFA in water) : 33% solvent B (0.1% TFA in MeCN) at 2 min to 47% A : 53% B at 20 min. For FAP-P-GP-MMAE, the gradient was modified from 60% A : 40% B at 2 min to 40% A : 60% B at 20 min.

Mass spectrometric analysis was performed on a TripleTOF system (SCIEX, USA) equipped with an electrospray ionization source operating in positive ion mode. Full-scan mass spectra were acquired in centroid format over the *m/z* range of 200-2000, with detector maximum count rates optimized for each specific compound: 1.80 × 10⁷ cps for FAP-O-VC-MMAE, 2.00 × 10⁷ cps for FAP-O-GP-MMAE, and 3.11 × 10⁷ cps for FAP-P-GP-MMAE, to ensure optimal detector response while preventing signal saturation.


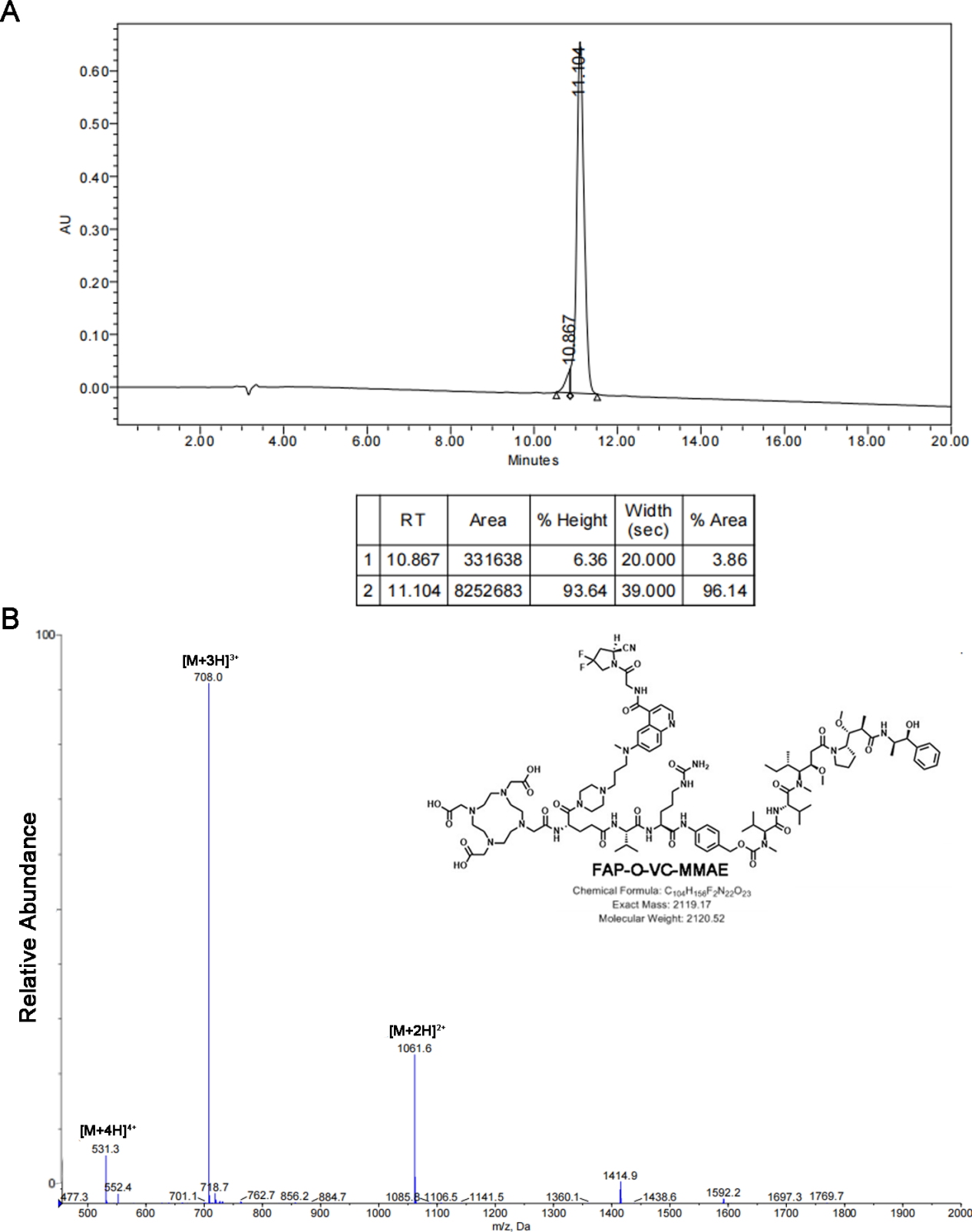


****Figure S1.** Analytical characterization of FAP‑O‑VC‑MMAE.** (**A**) HPLC chromatogram (PDA detection at 220 nm) showing a single peak at a retention time of 11.104 min, indicating high chemical purity. (**B**) High‑resolution mass spectrum (ESI‑positive mode). The observed signals at *m/z* 708.0 (triply charged, [M + 3H]^3+^), 1061.6 (doubly charged, [M + 2H]^2+^), and 531.3 (quadruply charged, [M + 4H]^4+^) correspond to the molecular mass of FAP‑O‑VC‑MMAE (calculated [M] = 2120.52 Da), confirming the identity and integrity of the synthesized conjugate.


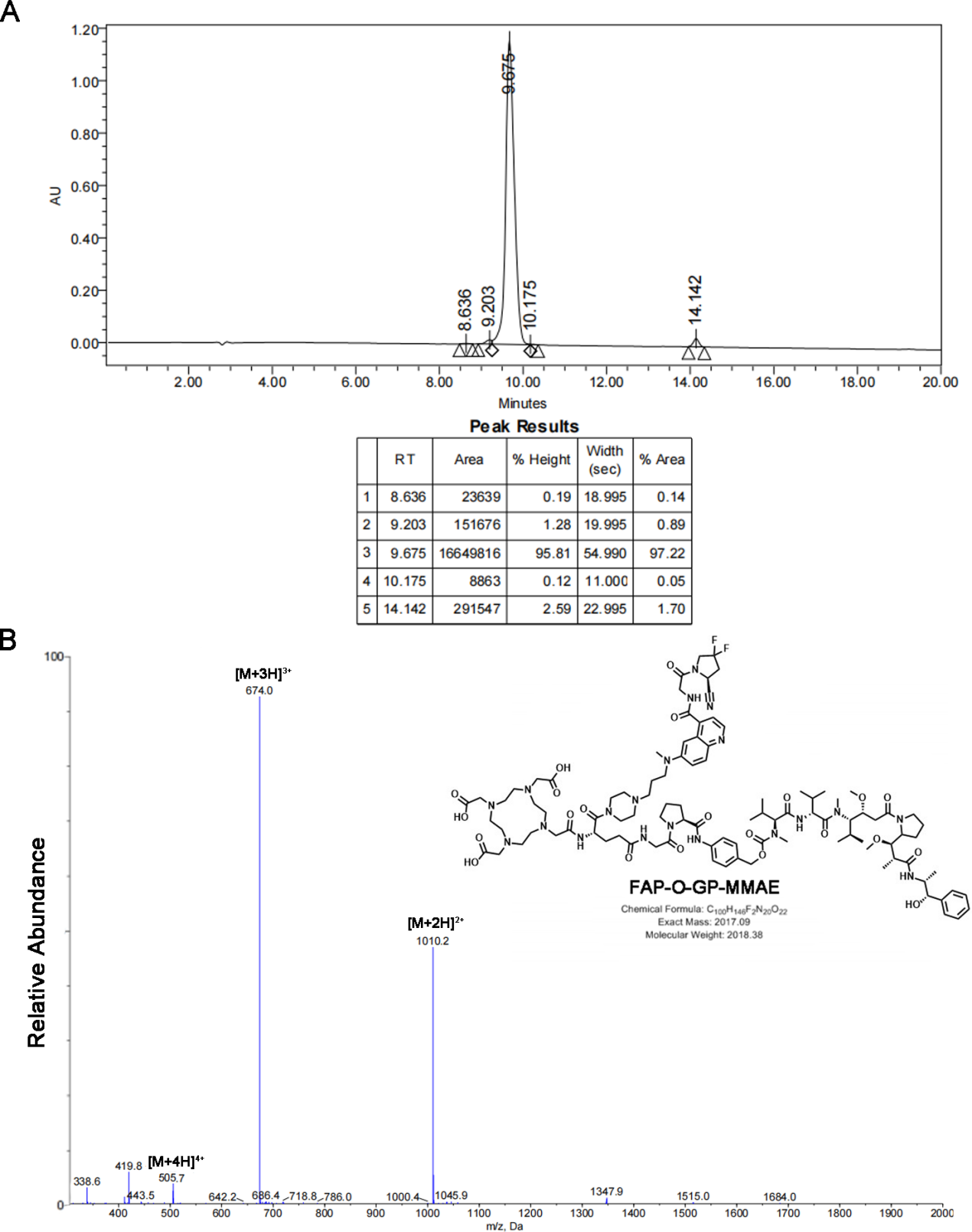


**Figure S2.** Analytical characterization of FAP‑O‑GP‑MMAE. (A) HPLC chromatogram (PDA detection at 220 nm) showing a single peak at a retention time of 9.675 min, indicating high chemical purity. (B) High‑resolution mass spectrum (ESI‑positive mode). The observed signals at *m/z* 674.0 (triply charged, [M + 3H]^3+^), 1010.2 (doubly charged, [M + 2H]^2+^), and 505.7 (quadruply charged, [M + 4H]^4+^) correspond to the molecular mass of FAP‑O‑GP‑MMAE (calculated [M] = 2118.38 Da), confirming the identity and integrity of the synthesized conjugate.


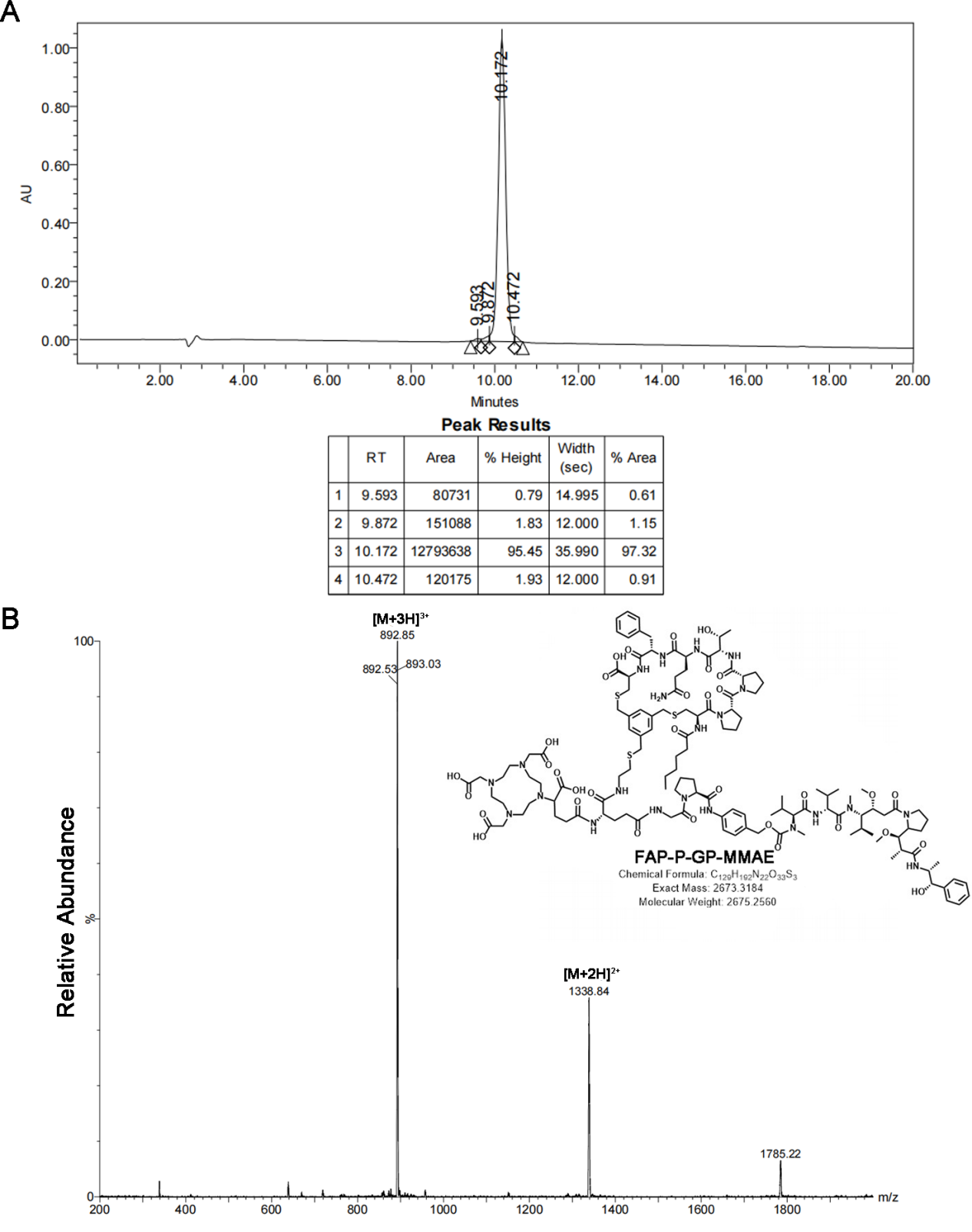


**Figure S3.** Analytical characterization of FAP‑P‑GP‑MMAE. (A) HPLC chromatogram (PDA detection at 220 nm) showing a single peak at a retention time of 10.172 min, demonstrating high chemical purity. (B) High‑resolution mass spectrum (ESI‑positive mode). The observed signals at *m/z* 892.85 (triply charged, [M + 3H]^3+^) and 1338.84 (doubly charged, [M + 2H]^2+^) correspond to the molecular mass of FAP‑P‑GP‑MMAE (calculated [M] = 2675.26 Da), confirming the identity and structural integrity of the synthesized conjugate.


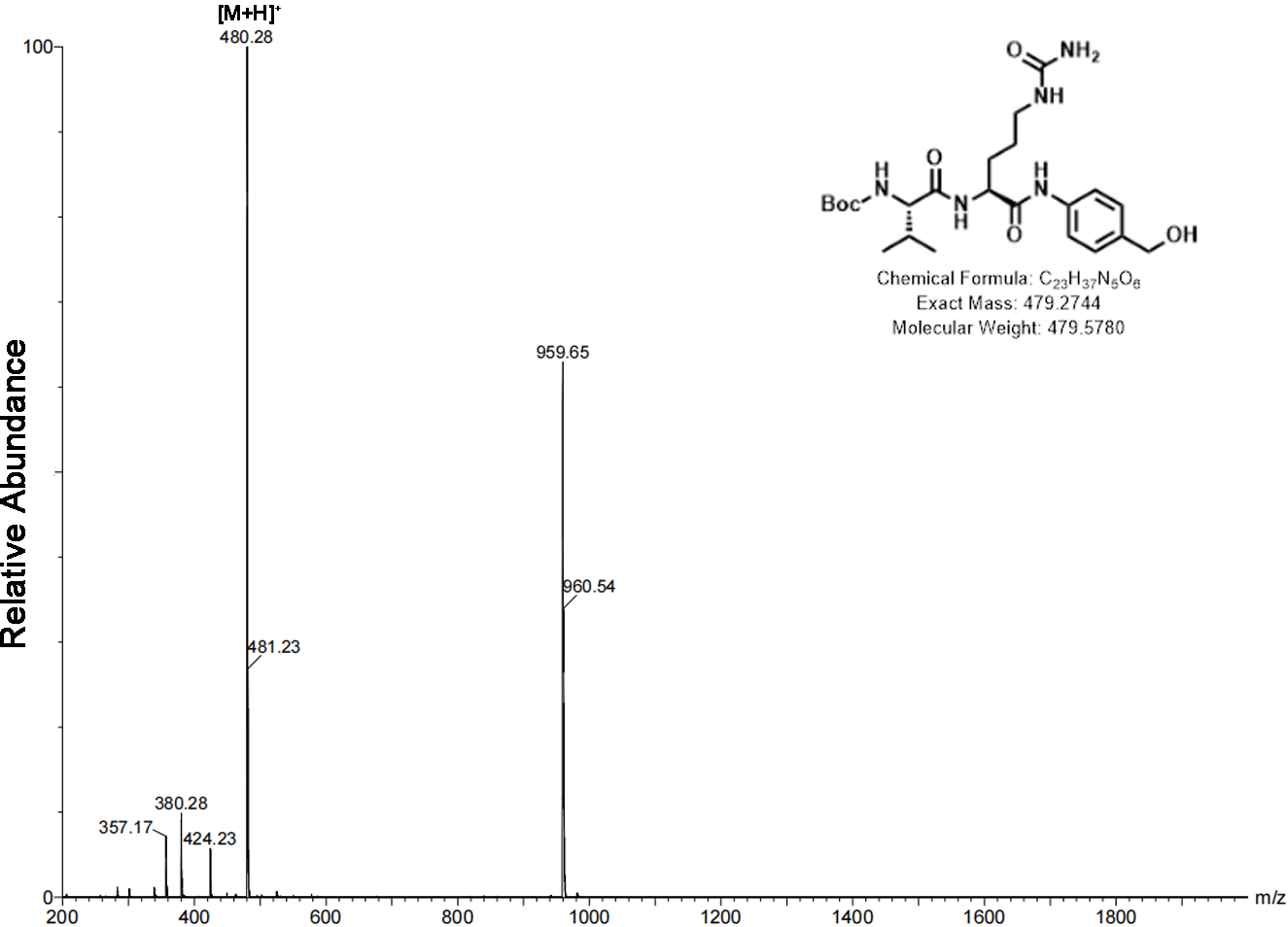


**Figure S4.** Mass spectrometric analysis of intermediate compound 4 (precursor to FAP-O-VC-MMAE). ESI-MS spectrum acquired in positive ion mode showing the characteristic multiply-charged ion pattern consistent with the expected molecular formula. The observed major peaks correspond to the singly protonated species [M + H]^+^ at *m/z* 480.28, which align with the calculated average isotopic mass of 479.27 Da for C_23_H_37_N_5_O_6_. The spectral pattern confirms the successful synthesis and identity of this key intermediate.


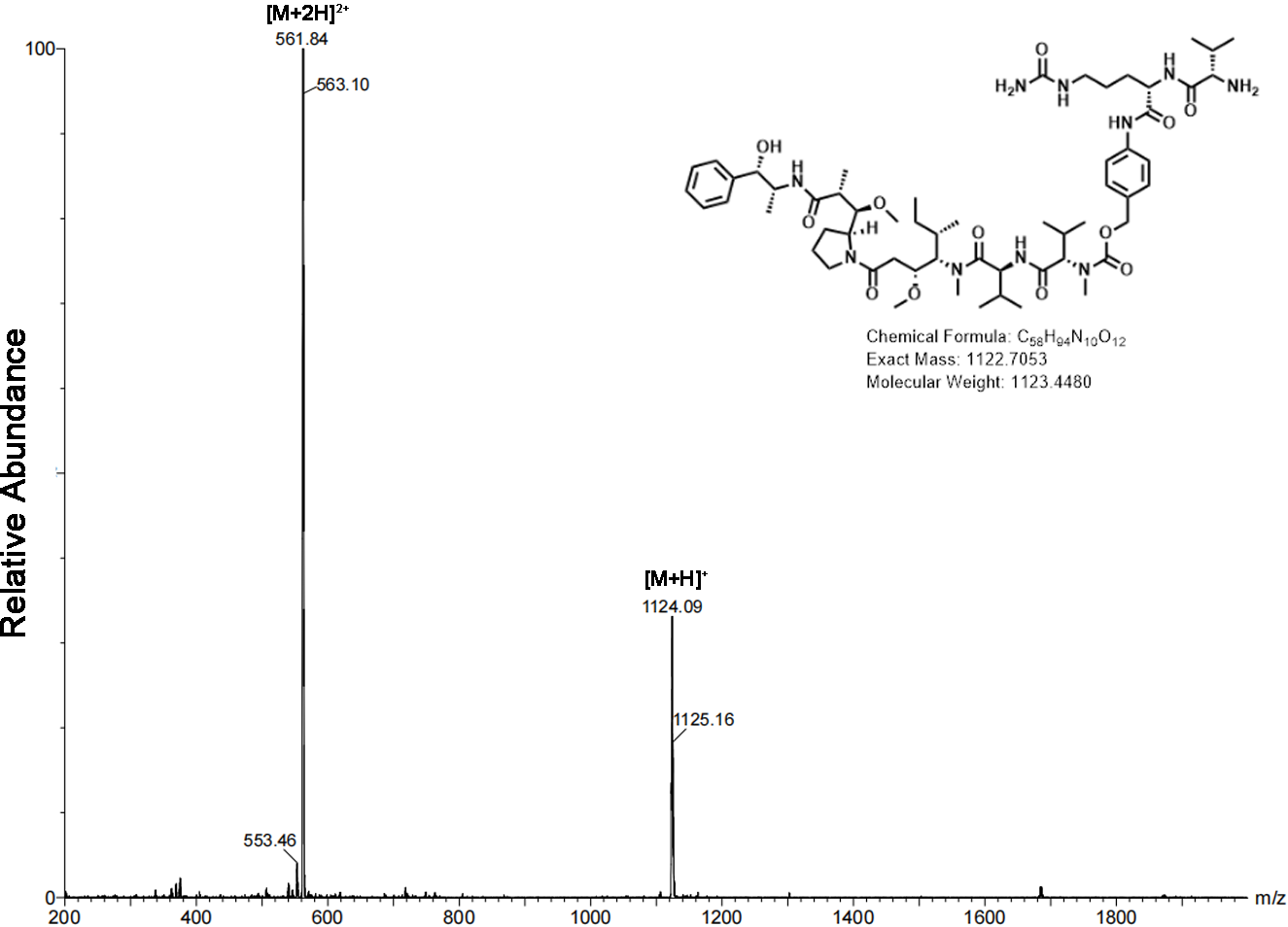


**Figure S5.** Mass spectrometric analysis of intermediate compound 6 (precursor to FAP-O-VC-MMAE). ESI-MS spectrum acquired in positive ion mode showing the characteristic multiply-charged ion pattern consistent with the expected molecular formula. The observed major peaks correspond to the singly protonated species [M + H]^+^ at *m/z* 1124.09 and the doubly protonated species [M + 2H]^2+^ at *m/z* 561.84, which align with the calculated average isotopic mass of 1122.71 Da for C_58_H_94_N_10_O_12_. The spectral pattern confirms the successful synthesis and identity of this key intermediate.


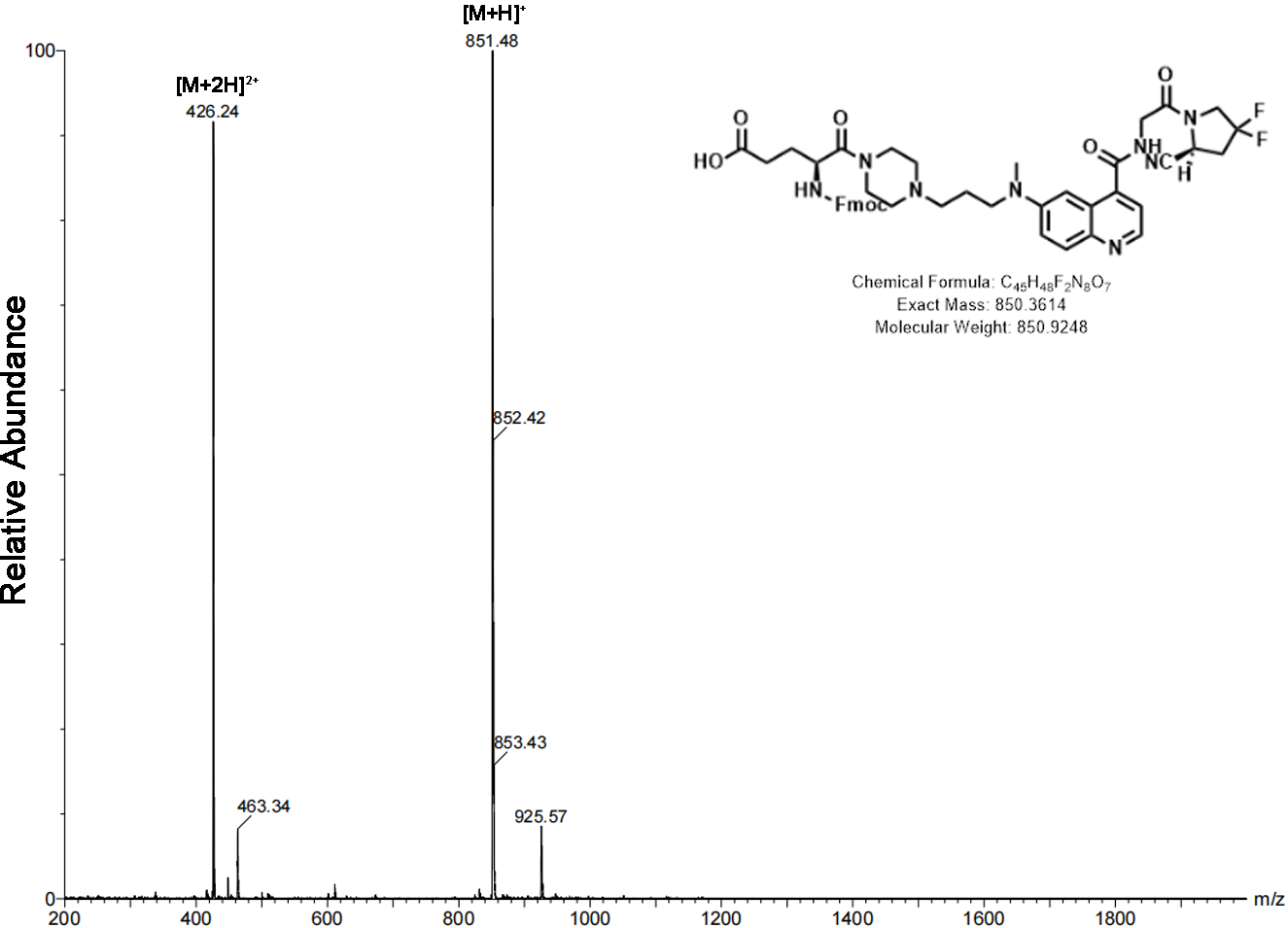


**Figure S6.** Mass spectrometric analysis of intermediate compound 9 (precursor to FAP-O-VC-MMAE). ESI-MS spectrum acquired in positive ion mode showing the characteristic multiply-charged ion pattern consistent with the expected molecular formula. The observed major peaks correspond to the singly protonated species [M + H]^+^ at *m/z* 851.48 and the doubly protonated species [M + 2H]^2+^ at *m/z* 426.24, which align with the calculated average isotopic mass of 850.36 Da for C_45_H_48_F_2_N_8_O_7_. The spectral pattern confirms the successful synthesis and identity of this key intermediate.


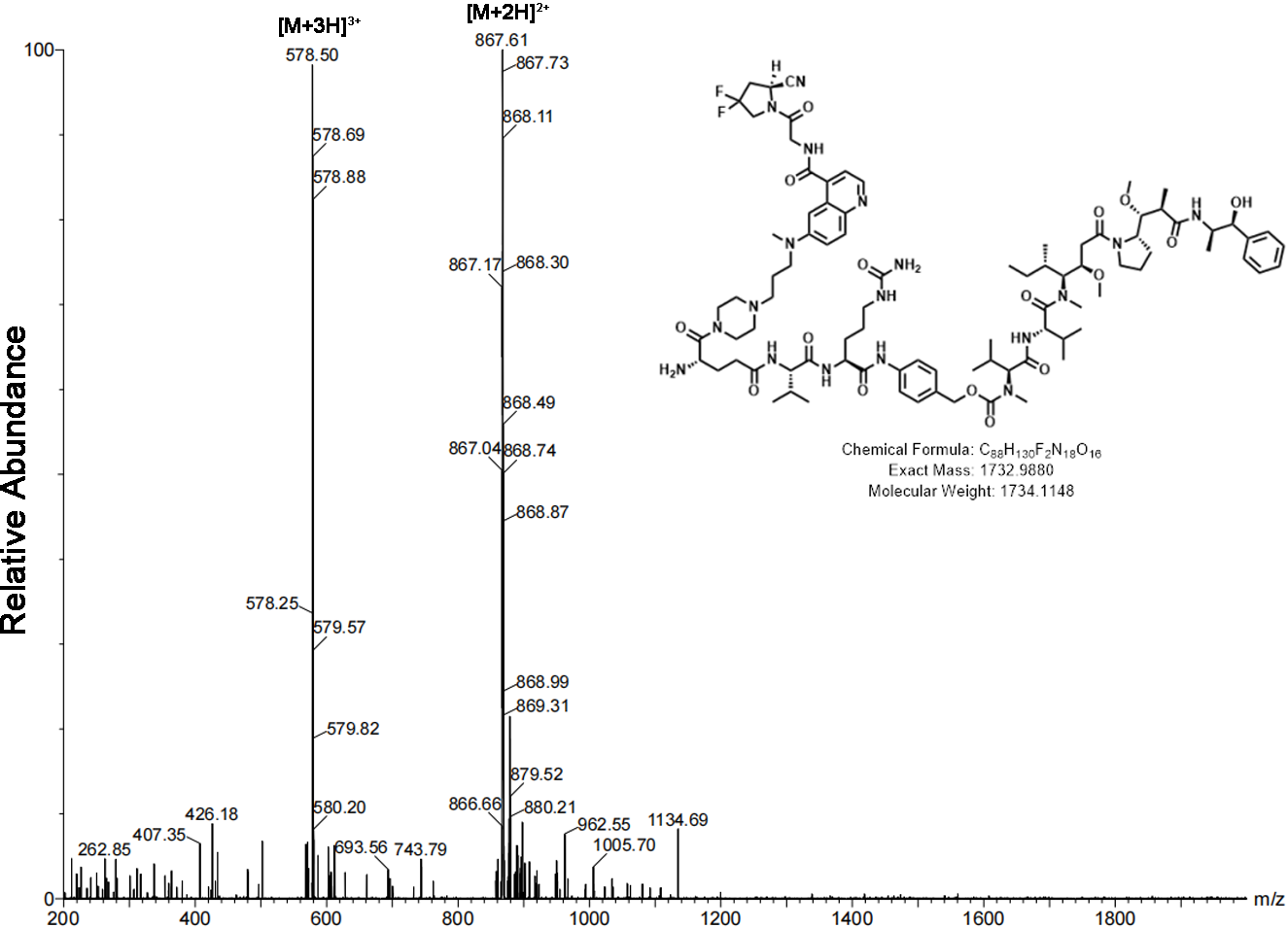


**Figure S7.** Mass spectrometric analysis of intermediate compound 10 (precursor to FAP-O-VC-MMAE). ESI-MS spectrum acquired in positive ion mode showing the characteristic multiply-charged ion pattern consistent with the expected molecular formula. The observed major peaks correspond to the doubly protonated species [M + H]^2+^ at *m/z* 867.61 and the triply protonated species [M + 3H]^3+^ at *m/z* 578.50, which align with the calculated average isotopic mass of 1732.99 Da for C_77_H_130_F_2_N_18_O_16_. The spectral pattern confirms the successful synthesis and identity of this key intermediate.

***
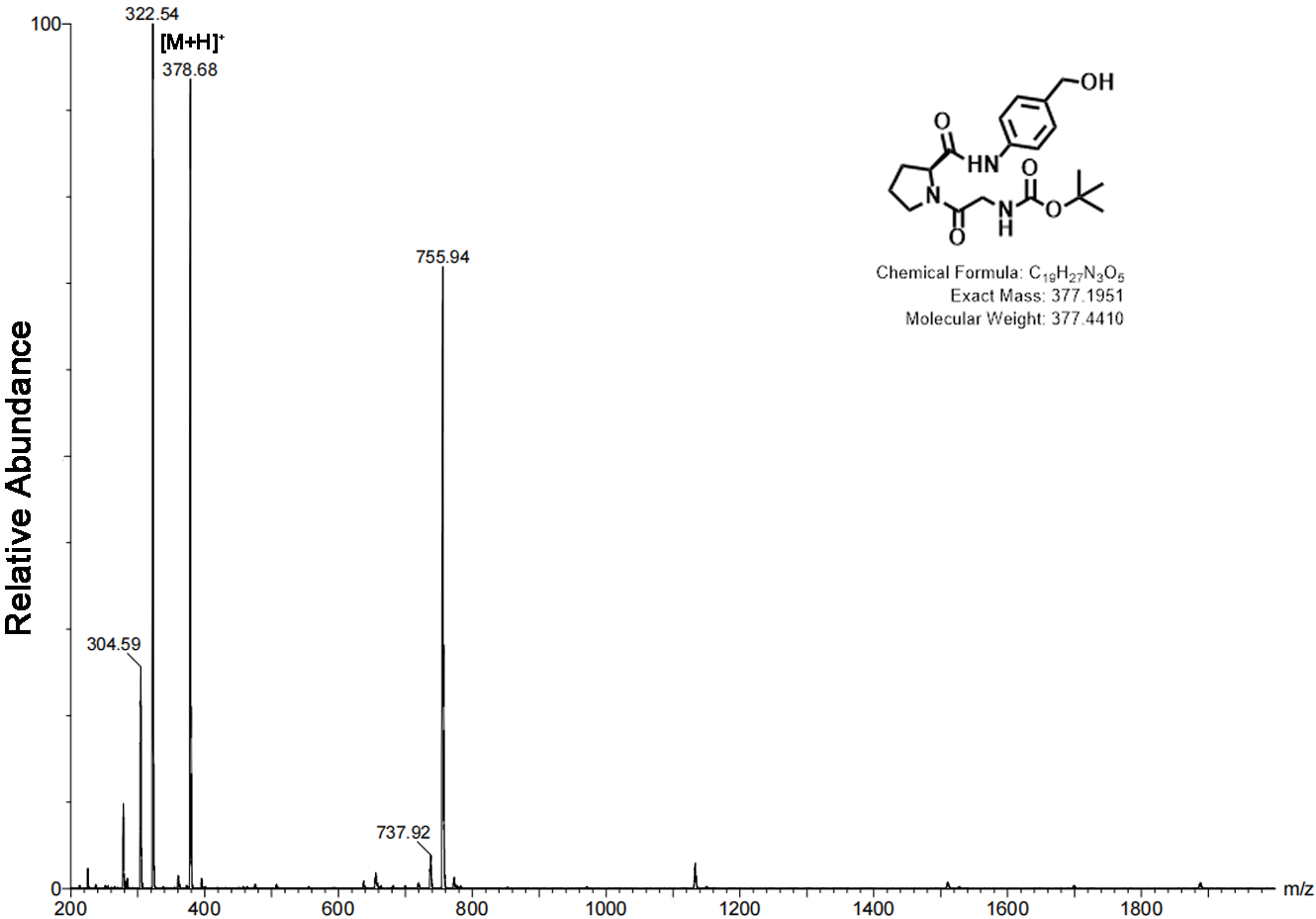
***

**Figure S8.** Mass spectrometric analysis of intermediate compound 13 (precursor to FAP-O-GP-MMAE). ESI-MS spectrum acquired in positive ion mode showing the characteristic multiply-charged ion pattern consistent with the expected molecular formula. The observed major peaks correspond to the singly protonated species [M + H]^+^ at *m/z* 378.68, which align with the calculated average isotopic mass of 377.20 Da for C_19_H_27_N_3_O_5_. The spectral pattern confirms the successful synthesis and identity of this key intermediate.

***
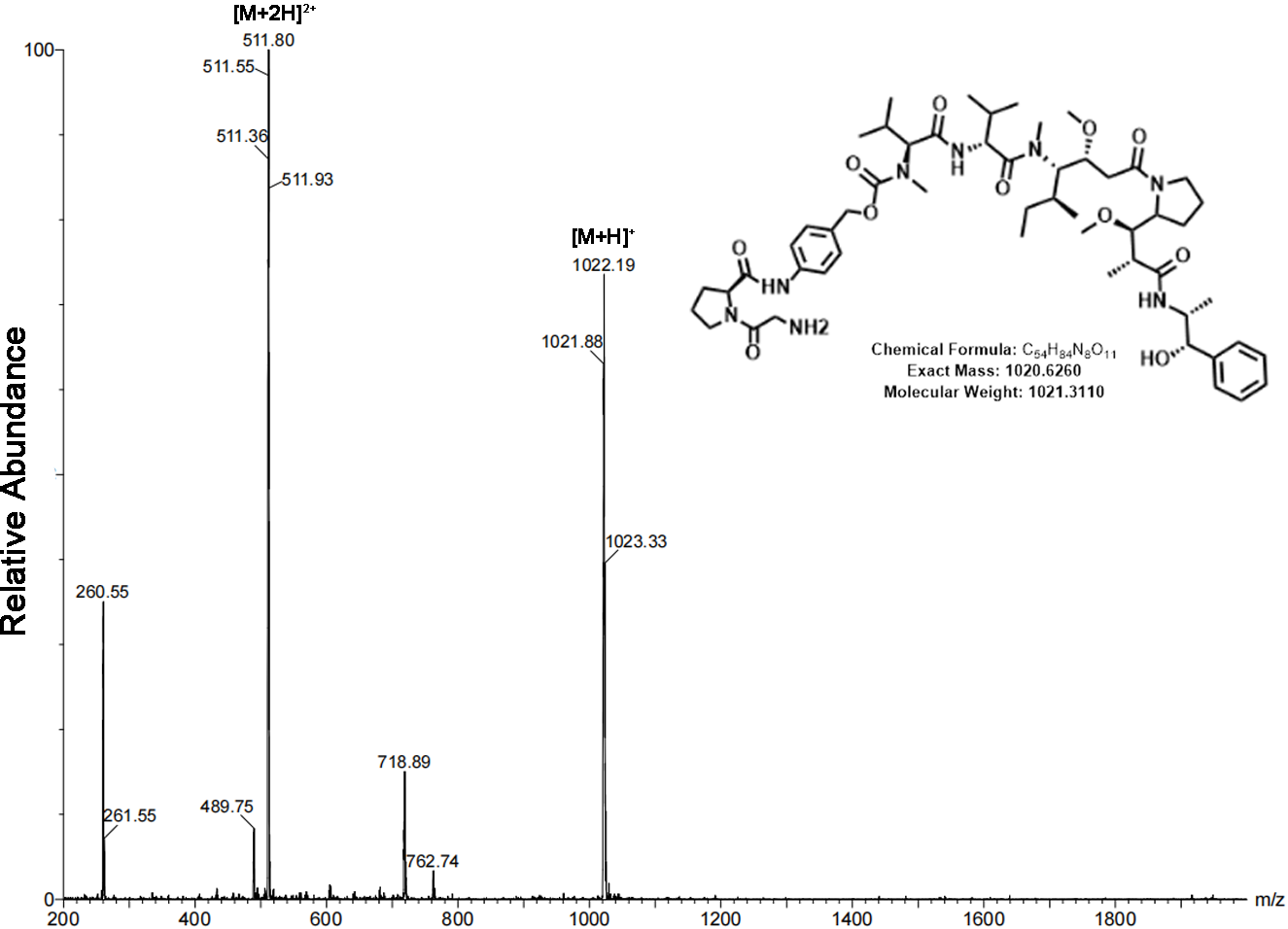
***

**Figure S9.** Mass spectrometric analysis of intermediate compound 14 (precursor to FAP-O-GP-MMAE). ESI-MS spectrum acquired in positive ion mode showing the characteristic multiply-charged ion pattern consistent with the expected molecular formula. The observed major peaks correspond to the singly protonated species [M + H]^+^ at *m/z* 1022.19 and the doubly protonated species [M + 2H]^2+^ at *m/z* 511.80, which align with the calculated average isotopic mass of 1020.63 Da for C_54_H_84_N_8_O_11_. The spectral pattern confirms the successful synthesis and identity of this key intermediate.

***
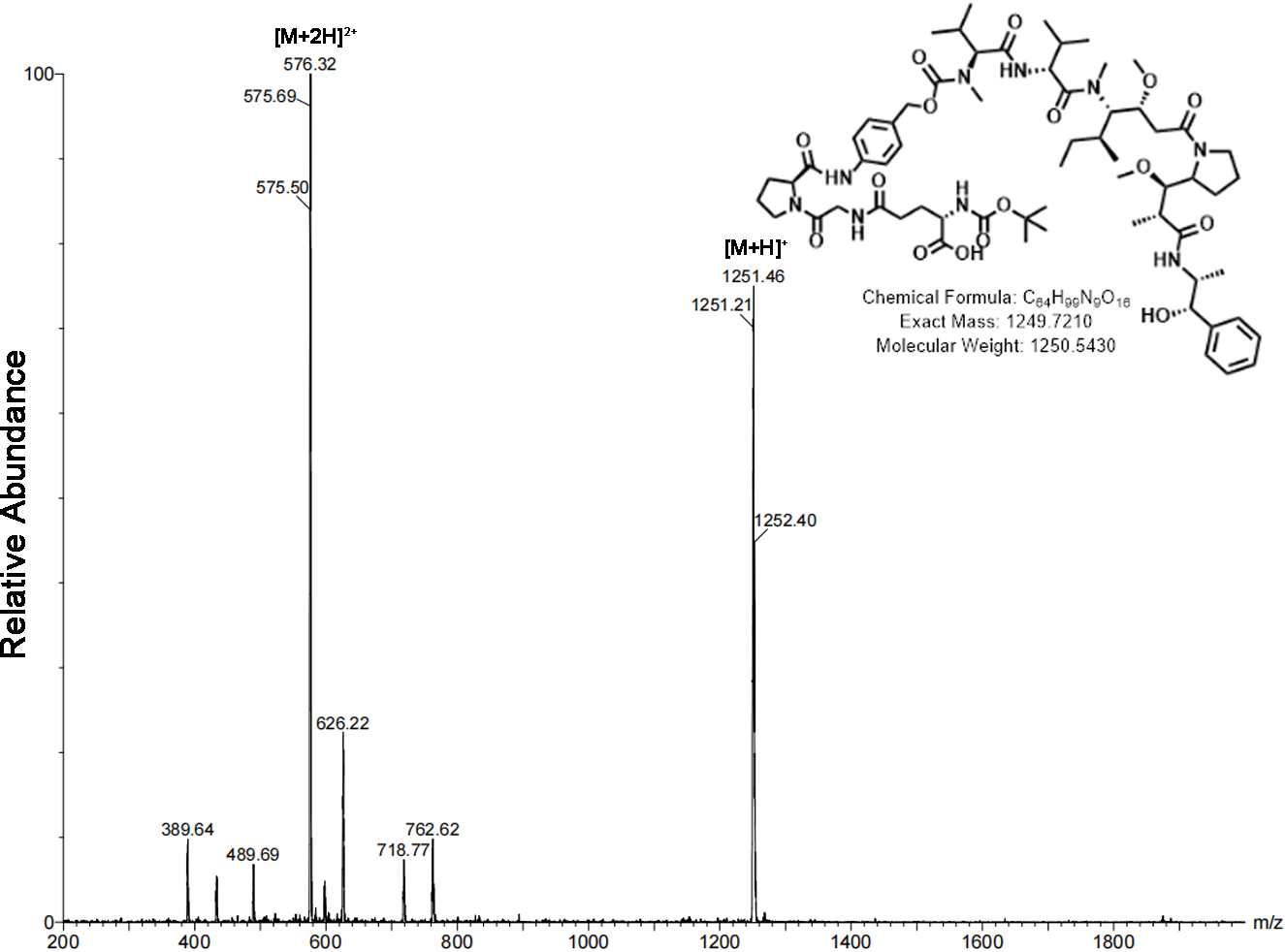
***

**Figure S10.** Mass spectrometric analysis of intermediate compound 16 (precursor to FAP-O-GP-MMAE). ESI-MS spectrum acquired in positive ion mode showing the characteristic multiply-charged ion pattern consistent with the expected molecular formula. The observed major peaks correspond to the singly protonated species [M + H]^+^ at *m/z* 1251.46 and the doubly protonated species [M + 2H]^2+^ at *m/z* 576.32, which align with the calculated average isotopic mass of 1249.72 Da for C_64_H_99_N_9_O_16_. The spectral pattern confirms the successful synthesis and identity of this key intermediate.

***
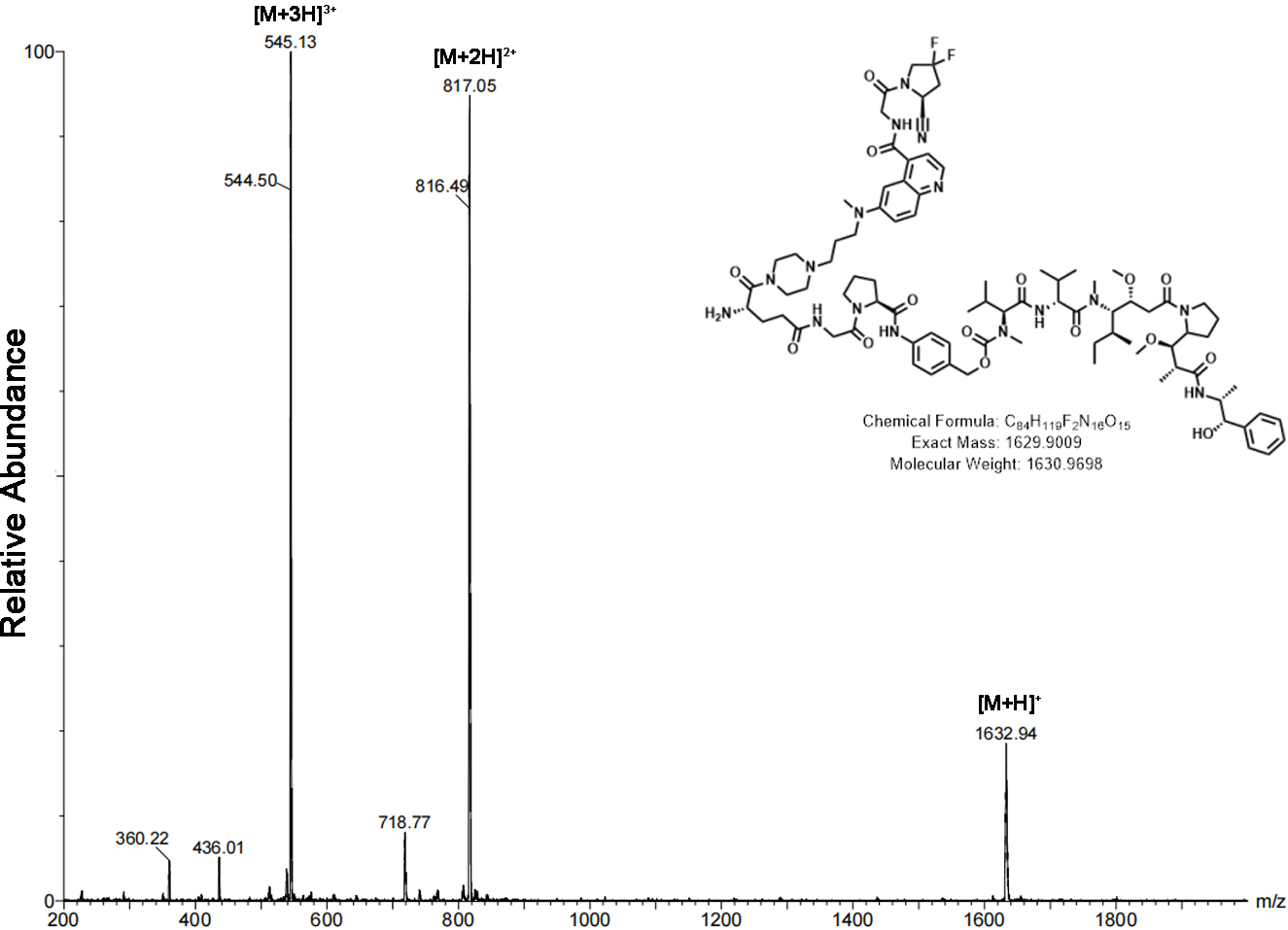
***

**Figure S11.** Mass spectrometric analysis of intermediate compound 17 (precursor to FAP-O-GP-MMAE). ESI-MS spectrum acquired in positive ion mode showing the characteristic multiply-charged ion pattern consistent with the expected molecular formula. The observed major peaks correspond to the singly protonated species [M + H]^+^ at *m/z* 1632.94, the doubly protonated species [M + 2H]^2+^ at *m/z* 817.05 and the triply protonated species [M + 3H]^3+^ at *m/z* 545.13, which align with the calculated average isotopic mass of 1629.90 Da for C_84_H_119_F_2_N_16_O_15_. The spectral pattern confirms the successful synthesis and identity of this key intermediate.

***
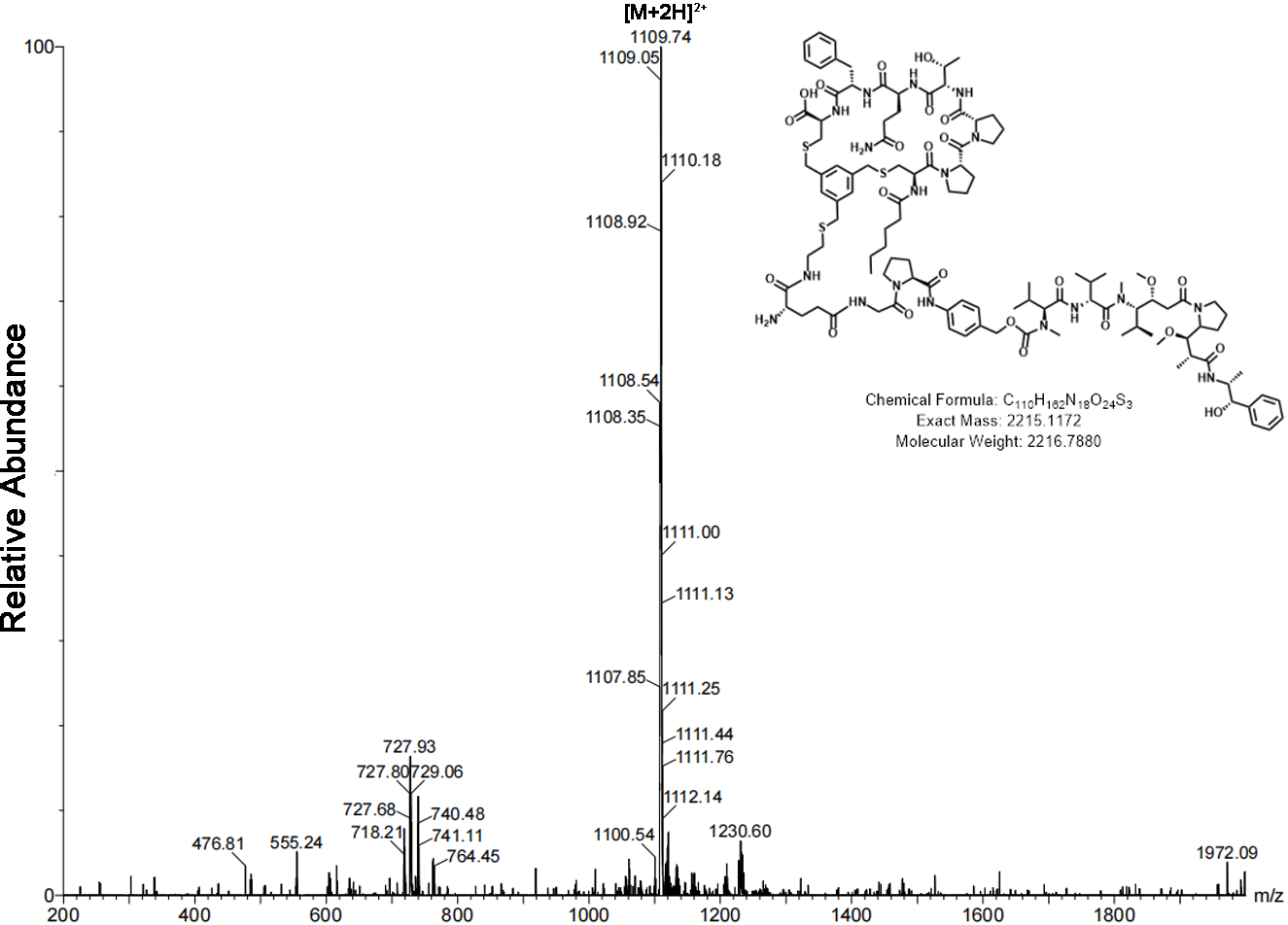
***

**Figure S12.** Mass spectrometric analysis of intermediate compound 19 (precursor to FAP-P-GP-MMAE). ESI-MS spectrum acquired in positive ion mode showing the characteristic multiply-charged ion pattern consistent with the expected molecular formula. The observed major peaks correspond to the doubly protonated species [M + H]^2+^ at *m/z* 1109.74, which align with the calculated average isotopic mass of 2215.12 Da for C_110_H_182_N_18_O_24_S_3_. The spectral pattern confirms the successful synthesis and identity of this key intermediate.

1. **Supporting Results**
   1. ***In Vitro Cathepsin B Cleavage Assay***


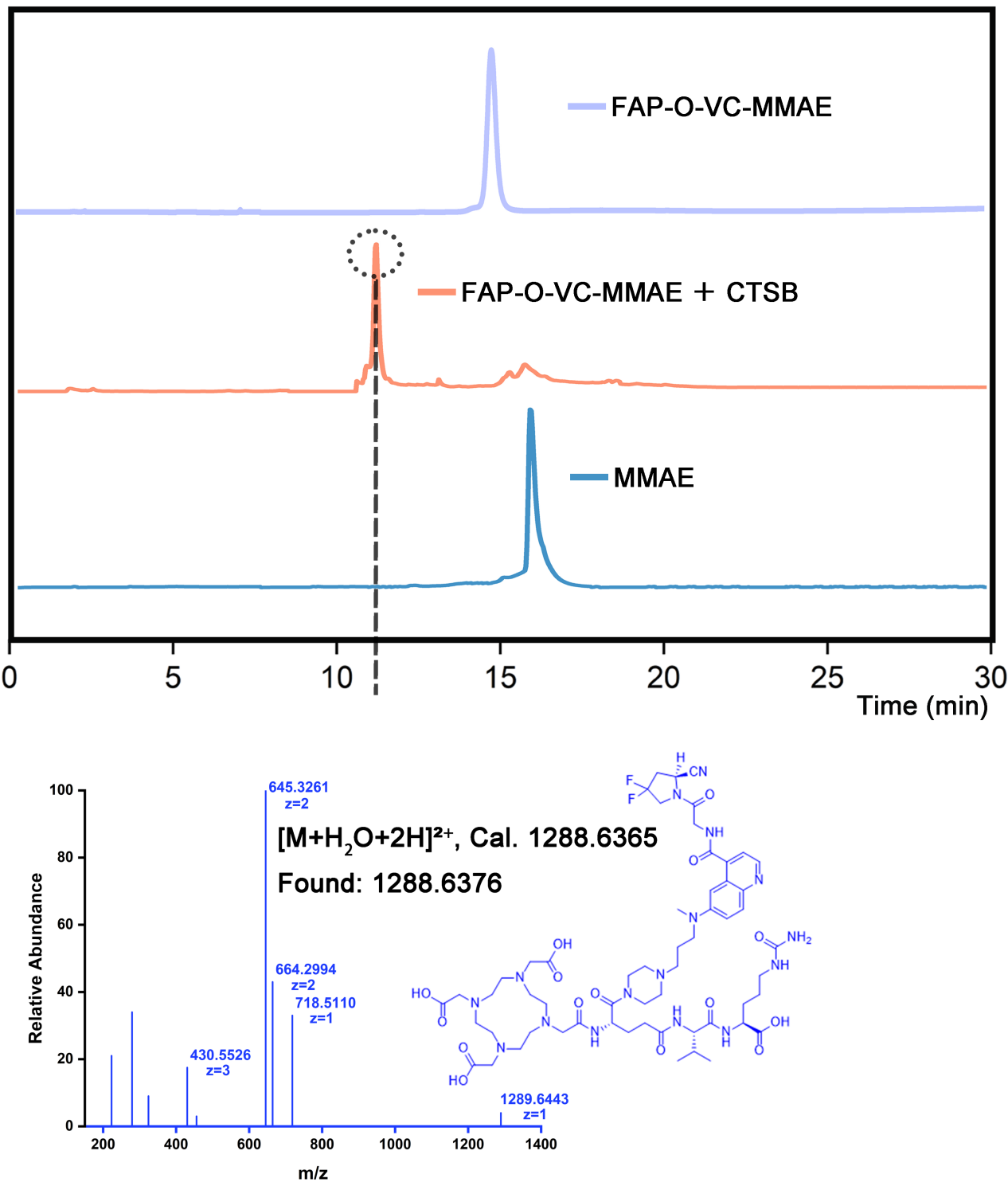


****Figure S13.** HRMS analysis of the putative FAP‑O‑VC‑DOTA fragment from CathB‑mediated cleavage of FAP‑O‑VC‑MMAE.** High‑resolution mass spectrum (ESI‑positive mode) of the early‑eluting product peak. The observed ions at *m/z* 645.3261 (assigned as [M + 2H]^2+^) and 1289.6443 (assigned as [M + H]^+^) align with the theoretical mass of the expected FAP‑O‑VC‑DOTA moiety bearing one water molecule adduct (calculated for C_57_H_84_F_2_N_16_O_15_ + H₂O: [M + H₂O + H]⁺ = 1289.6450, mass error <1 ppm). These data are consistent with the formation of this fragment upon CathB cleavage.

*2.2 Individual Tumor Growth Curves from the In Vivo Therapy Study*


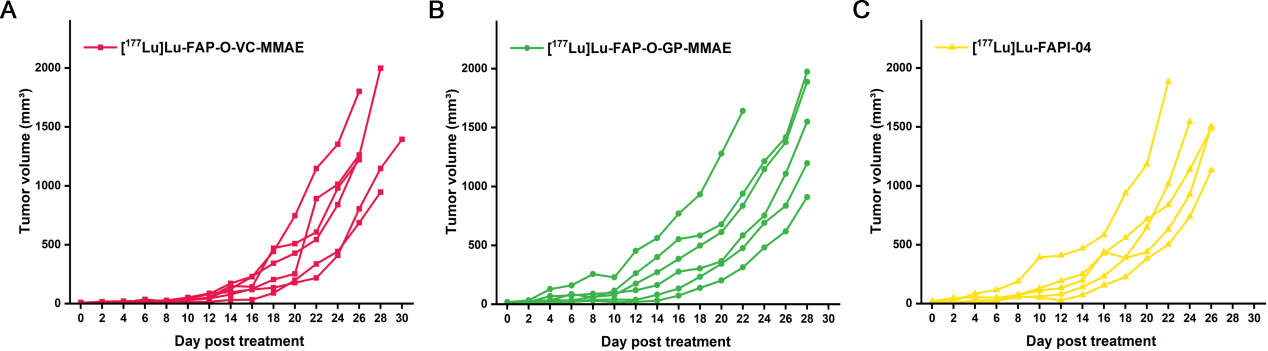


**Figure S14.** Individual tumor growth curves for each animal in the radionuclide monotherapy groups from the U87MG xenograft efficacy study. (A) Group 1: [¹⁷⁷Lu]Lu-FAP‑O‑VC‑MMAE (n = 6). (B) Group 2: [¹⁷⁷Lu]Lu-FAP‑O‑GP‑MMAE (n = 6). (C) Group 3: [¹⁷⁷Lu]Lu-FAPI‑04 (n = 5). Tumor volume change over time for each mouse is plotted individually.


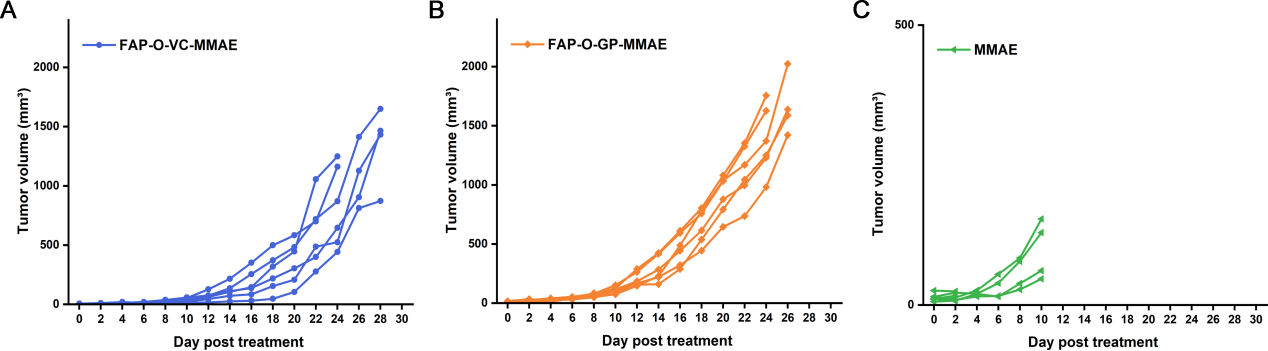


**Figure S15.** Individual tumor growth curves for each animal in the chemotherapy-only groups from the U87MG xenograft efficacy study. (A) Group 4: FAP‑O‑VC‑MMAE (n = 6). (B) Group 5: FAP‑O‑GP‑MMAE (n = 6). (C) Group 6: MMAE (n = 8). Tumor volume change over time for each mouse is plotted individually.


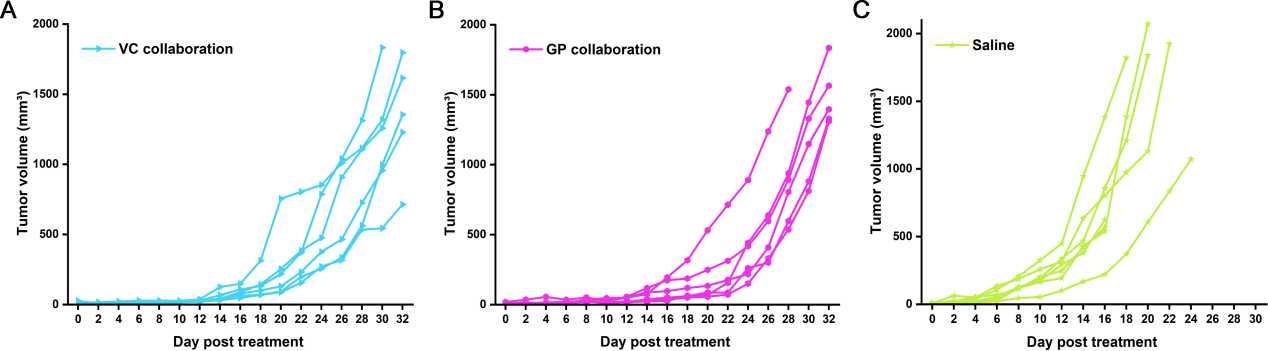


**Figure S16.** Individual tumor growth curves for each animal in the combination therapy and control groups from the U87MG xenograft efficacy study. (A) Group 7: VC combination therapy (n = 6). (B) Group 8: GP combination therapy (n = 6). (C) Group 9: Saline control (n = 6). Tumor volume change over time for each mouse is plotted individually.

*2.3 Tumor growth inhibition (TGI) analysis of all treatment groups.*


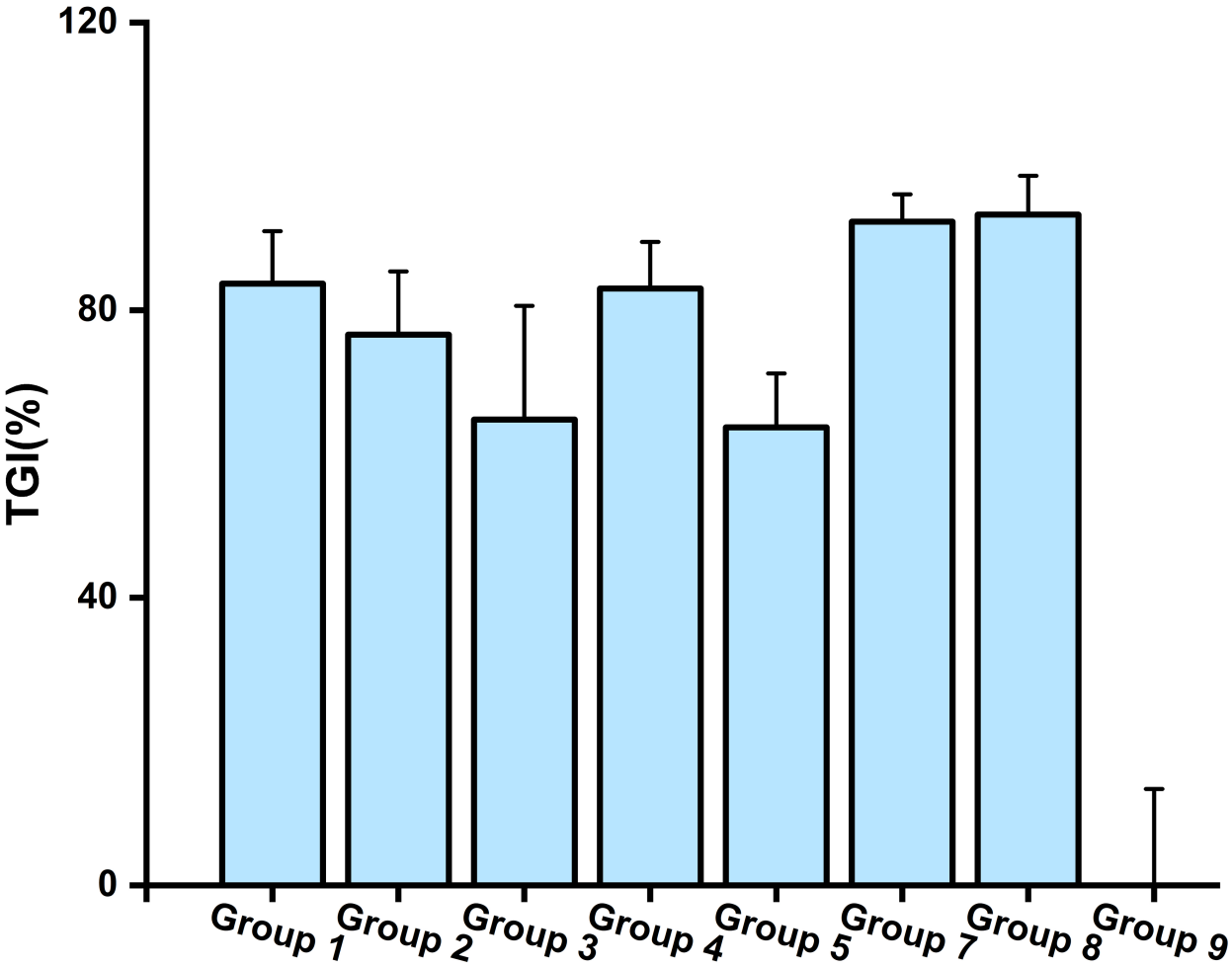


**Figure S17.** Tumor growth inhibition (TGI) rates of all treatment groups on day 20, including radionuclide monotherapy (Groups 1–2), SMDC monotherapy (Groups 4–5), combination therapy (Groups 7–8), and control groups (Groups 3, 6, and 9). These data complement the therapeutic evaluation presented in Figure 7. The separation of TGI and survival analyses improves clarity of data presentation in the main figure.

**References**

1. S. Peng, B. Li, M. Sun, J. Yang, Z. Cai, Y. Liu, P. Tang, D. Feng, Q. Cao, P. Ran, K. Hu, “Development of Cysteic Acid-Modified FAP Radioligands for Enhanced Renal Clearance: From Preclinical Optimization to First-in-Human Study,” *J Med Chem* **68** (2025): 14019-14027, https://doi.org/10.1021/acs.jmedchem.5c01163.
2. Y. Liu, P. Tang, S. Peng, J. Zhong, Z. Xu, J. Zhong, J. Su, Y. Zhong, K. Hu, “[^18^F]AlF-CBP imaging of type I collagen for non-invasive monitoring of pulmonary fibrosis in preclinical models,” *Eur J Nucl Med Mol Imaging* **52** (2024): 22-35, https://doi.org/10.1007/s00259-024-06888-3.
3. K. Hu, J. Li, L. Wang, Y. Huang, L. Li, S. Ye, Y. Han, S. Huang, H. Wu, J. Su, G. Tang, “Preclinical evaluation and pilot clinical study of [^18^F]AlF-labeled FAPI-tracer for PET imaging of cancer associated fibroblasts,” *Acta Pharm Sin B* **12** (2022): 867-875, https://doi.org/10.1016/j.apsb.2021.09.032.
4. Q. Yan, J. Zhong, Y. Liu, S. Peng, P. Feng, Y. Zhong, K. Hu, “Synthesis and preclinical evaluation of a heterodimeric radioligand targeting fibroblast activation protein and integrin-α_v_β_3_,” *Eur J Med Chem*, **251** (2023): 115279, https://doi.org/10.1016/j.ejmech.2023.115279.
